# Reversible transitions between noradrenergic and mesenchymal tumor identities define cell plasticity in neuroblastoma

**DOI:** 10.1038/s41467-023-38239-5

**Published:** 2023-05-04

**Authors:** Cécile Thirant, Agathe Peltier, Simon Durand, Amira Kramdi, Caroline Louis-Brennetot, Cécile Pierre-Eugène, Margot Gautier, Ana Costa, Amandine Grelier, Sakina Zaïdi, Nadège Gruel, Irène Jimenez, Eve Lapouble, Gaëlle Pierron, Déborah Sitbon, Hervé J. Brisse, Arnaud Gauthier, Paul Fréneaux, Sandrine Grossetête, Laura G. Baudrin, Virginie Raynal, Sylvain Baulande, Angela Bellini, Jaydutt Bhalshankar, Angel M. Carcaboso, Birgit Geoerger, Hermann Rohrer, Didier Surdez, Valentina Boeva, Gudrun Schleiermacher, Olivier Delattre, Isabelle Janoueix-Lerosey

**Affiliations:** 1 Institut Curie, Inserm U830, PSL Research University, Diversity and Plasticity of Childhood Tumors Lab, Paris, France; 2 SIREDO: Care, Innovation and Research for Children, Adolescents and Young Adults with Cancer, Institut Curie, Paris, France; 3 Institut Curie, Department of Translational Research, Paris, France; 4 Institut Curie, Laboratoire Recherche Translationnelle en Oncologie Pédiatrique (RTOP), Laboratoire “Gilles Thomas”, Paris, France; 5 Institut Curie, Unité de Génétique Somatique, Paris, France; 6 Institut Curie, Department of Imaging, PSL Research University, Paris, France; 7 Institut Curie, Department of Biopathology, Paris, France; 8 Institut Curie, Genomics of Excellence (ICGex) Platform, Paris, France. Institut Curie, Single Cell Initiative, Paris, France; 9 SJD Pediatric Cancer Center Barcelona, Institut de Recerca Sant Joan de Déu, Barcelona, Spain; 10 Gustave Roussy Cancer Campus, INSERM U1015, Department of Pediatric and Adolescent Oncology, Université Paris-Saclay, Villejuif, France; 11 Institute of Clinical Neuroanatomy, Dr. Senckenberg Anatomy, Neuroscience Center, Goethe University, Frankfurt/M, Germany; 12 Balgrist University Hospital, Faculty of Medicine, University of Zurich (UZH), Zurich, Switzerland; 13 Inserm, U1016, Cochin Institute, CNRS UMR8104, Paris University, Paris, France; 14 ETH Zürich, Department of Computer Science, Institute for Machine Learning, Zürich, Switzerland; 15 Swiss Institute of Bioinformatics (SIB), Zürich, Switzerland

**Keywords:** Paediatric cancer, Epigenetics, CNS cancer, Cancer genomics, Tumour heterogeneity

## Abstract

Noradrenergic and mesenchymal identities have been characterized in neuroblastoma cell lines according to their epigenetic landscapes and core regulatory circuitries. However, their relationship and relative contribution in patient tumors remain poorly defined. We now document spontaneous and reversible plasticity between the two identities, associated with epigenetic reprogramming, in several neuroblastoma models. Interestingly, xenografts with cells from each identity eventually harbor a noradrenergic phenotype suggesting that the microenvironment provides a powerful pressure towards this phenotype. Accordingly, such a noradrenergic cell identity is systematically observed in single-cell RNA-seq of 18 tumor biopsies and 15 PDX models. Yet, a subpopulation of these noradrenergic tumor cells presents with mesenchymal features that are shared with plasticity models, indicating that the plasticity described in these models has relevance in neuroblastoma patients. This work therefore emphasizes that intrinsic plasticity properties of neuroblastoma cells are dependent upon external cues of the environment to drive cell identity.

## Abstract (web summary)

Noradrenergic and mesenchymal cell states have been proposed in neuroblastoma, but their contributions to the tumour are not clearly understood. Here, the authors used in vitro and in vivo models, as well as single-cell RNA-seq, to characterise noradrenergic and mesenchymal cells and their phenotypic plasticity in neuroblastoma.

## Introduction

Neuroblastoma is a childhood cancer arising from the peripheral sympathetic nervous system, known to be derived from multipotent neural crest cells (NCCs). The hallmark of neuroblastoma is its wide range of clinical presentations and outcomes, ranging from spontaneous regression to fatal outcome despite multimodal therapies^1^. High-risk neuroblastoma most often initially respond to intensive chemotherapy; however, relapses frequently occur followed by fatal outcome. Several genes including *MYCN*^2^, *ALK*^3–6^, and *TERT*^7–9^ have been identified as key drivers of neuroblastoma oncogenesis.

Neuroblastomas mostly develop in the adrenal gland but a subset of them originates from sympathetic ganglia along the paravertebral sympathetic chains^1^. With respect to these localizations, neuroblastoma likely arises from the transformation of sympathoblasts either in sympathetic ganglia or in the adrenal medulla, from catecholamine-secreting chromaffin cells of the adrenal medulla, or alternatively from a common sympatho-adrenal progenitor^10,11^. Several groups have recently tackled the cell of origin of neuroblastoma by comparing tumor cell transcriptomic profiles to developmental transcriptomic profiles of human embryonic and fetal adrenal medulla at the single-cell level^12–16^. However, no consensus has been reached so far, underlying the need to extend our knowledge on this heterogeneous disease. The master transcriptional regulators controlling the gene expression program of neuroblastoma have been highlighted through the characterization of the super-enhancer landscape of neuroblastoma cell lines, revealing two distinct cell identities: a sympathetic noradrenergic identity defined by a core regulatory circuitry (CRC) module including the PHOX2A/B, HAND1/2 and GATA2/3 transcription factors and a NCC-like/mesenchymal identity, close to that of human neural crest cells (hNCCs), driven by factors of the AP1 family among others^17,18^. Additional transcription factors participating in the noradrenergic CRC have subsequently been characterized including ISL1, TBX2 and ASCL1^19–21^. Importantly, mesenchymal tumor cells in vitro have been shown to be more resistant to standard chemotherapy^17,18^ suggesting that they may be involved in therapeutic resistance and relapses in neuroblastoma patients. Bulk RNA-seq analyses or immunohistochemistry (IHC) with few markers suggested that mesenchymal tumor cells may be present in patient tumors and that some tumors exhibit a mesenchymal identity^17,18,22,23^. Cellular plasticity between the noradrenergic and mesenchymal states has been reported for a few cell lines^18,24–26^, but in most instances specific molecular markers were missing to sort the cells of each type and further investigate cell phenotype switch from one to the other state.

In this work, we use several cellular models, single-cell transcriptomics and epigenetic analyses to characterize the noradrenergic and mesenchymal identities and explore cell plasticity in neuroblastoma. We uncover and use CD44 as a surrogate marker of the mesenchymal identity in vitro. We document that a noradrenergic-to-mesenchymal transition can be promoted by growth factors such as EGF and TNFα in vitro whereas the mesenchymal-to-noradrenergic reprogramming is demonstrated in vivo. Finally, single-cell transcriptomic analyses on neuroblastoma biopsies and PDXs unveil intra-tumor heterogeneity and highlight a population of noradrenergic tumor cells expressing mesenchymal markers, reminiscent of the plasticity observed in cellular models.

## Results

### The cell surface marker CD44 discriminates noradrenergic and mesenchymal tumor cells in neuroblastoma heterogeneous cell lines

**Fig. 1:**
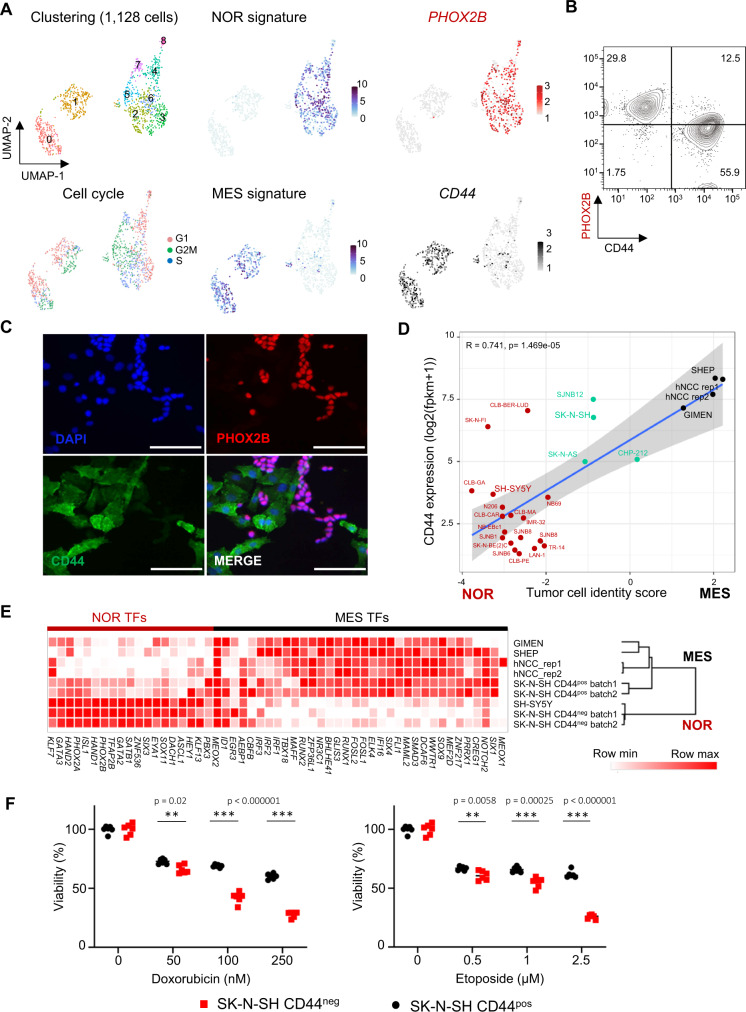
The cell surface marker CD44 discriminates noradrenergic and mesenchymal tumor cells in the SK-N-SH cell line. NOR noradrenergic, MES mesenchymal. **A** Single-cell RNA-seq analysis by Seurat of the SK-N-SH cell line (batch 1). The umap plot shows the clustering at resolution 0.8 and the cell cycle score. Two main cell identities are highlighted by noradrenergic and mesenchymal transcription factor signatures^17,18^ and *PHOX2B* and *CD44* expression, respectively. Each cell identity includes cycling cells. **B** FACS analysis of the SK-N-SH cell line (batch 1) after cell permeabilization using PHOX2B and CD44 antibodies, gated in live cells after doublet exclusion. **C** Immunofluorescence analysis of the SK-N-SH cell line (batch 1) with the PHOX2B and CD44 markers (scale bar = 50 µm, representative of 3 independent experiments). **D** Scatterplot showing the correlation of *CD44* expression by bulk RNAseq in each cell line with the tumor cell identity score (score MES - score NOR). Noradrenergic and mesenchymal scores correspond to the mean expression of transcription factors that define each identity. Simple linear regression line is shown, and gray cloud represents the 95% two-tailed confidence interval of the slope. The measure of linear association is given by Pearson’s product moment correlation production (r) with its associated p-value (*t* = 5.4123, df = 24, 95% CI for r [0.4966679, 0.8769103]). Color code: red = noradrenergic, black = mesenchymal, green =  intermediate cell lines. **E** Unsupervised clustering of samples using the expression of noradrenergic and mesenchymal transcription factors (TFs)^17,18^ on bulk RNAseq data indicates that CD44^neg^ and CD44^pos^ sorted cells exhibit a transcriptomic profile close to the noradrenergic SH-SY5Y and mesenchymal SH-EP, GIMEN or hNCC cells, respectively. Two independent replicates of SK-N-SH (batches 1 and 2) have been analyzed. **F** Mesenchymal/CD44^pos^ sorted cells are more resistant to doxorubicin and etoposide than noradrenergic/CD44^neg^ cells. Cell viability was measured by resazurin assay after 72 h of chemotherapy treatments (Doxorubicin 50, 100, 250 nM and Etoposide 0.5, 1, 2.5 µM) (mean ± sd; *n* = 6 replicates). *P*-values were determined via two-tailed unpaired Welch’s *t*-test. Source data are provided as a Source Data file.

Our previous study has shown that most of the neuroblastoma cell lines (18 out of 25) exhibit a noradrenergic identity whereas only 3 have a mesenchymal epigenetic profile. An intermediate group of cells expressing noradrenergic and mesenchymal transcription factors was composed of 4 samples, including the heterogeneous SK-N-SH cell line^17^. Interestingly, morphological heterogeneity has been reported for this cell line^24^. Single-cell transcriptomic sequencing with the 10X Genomics technology and analyses of previously described transcription factor signatures^17,18^ have now revealed the co-existence of two cell populations in SK-N-SH. Noradrenergic cells expressed *PHOX2B* whereas *CD44*^27–29^ appeared as a specific marker of the mesenchymal population, which was confirmed by FACS and immunofluorescence (Fig. 1A–C). Of note, *CD44* expression at transcription level correlates with mesenchymal tumor cell identity in neuroblastoma cell lines (Fig. 1D). As CD44 is a cell surface marker, it was further used to sort by FACS both populations. Bulk RNA-seq experiments confirmed that CD44^neg^ and CD44^pos^ sorted cells exhibited transcriptomic profiles close to the noradrenergic SH-SY5Y and mesenchymal SH-EP, GIMEN and hNCC cell lines, respectively (Fig. 1E). As expected from previous data obtained with the SH-SY5Y and SH-EP cell lines^17^, originally sub-cloned from the heterogeneous parental SK-N-SH cell line^24^, the mesenchymal/CD44^pos^ population of this cell line exhibited a higher resistance to standard chemotherapies used in clinics, as compared to the noradrenergic/CD44^neg^ cells (Fig. 1F).

**Fig. 2:**
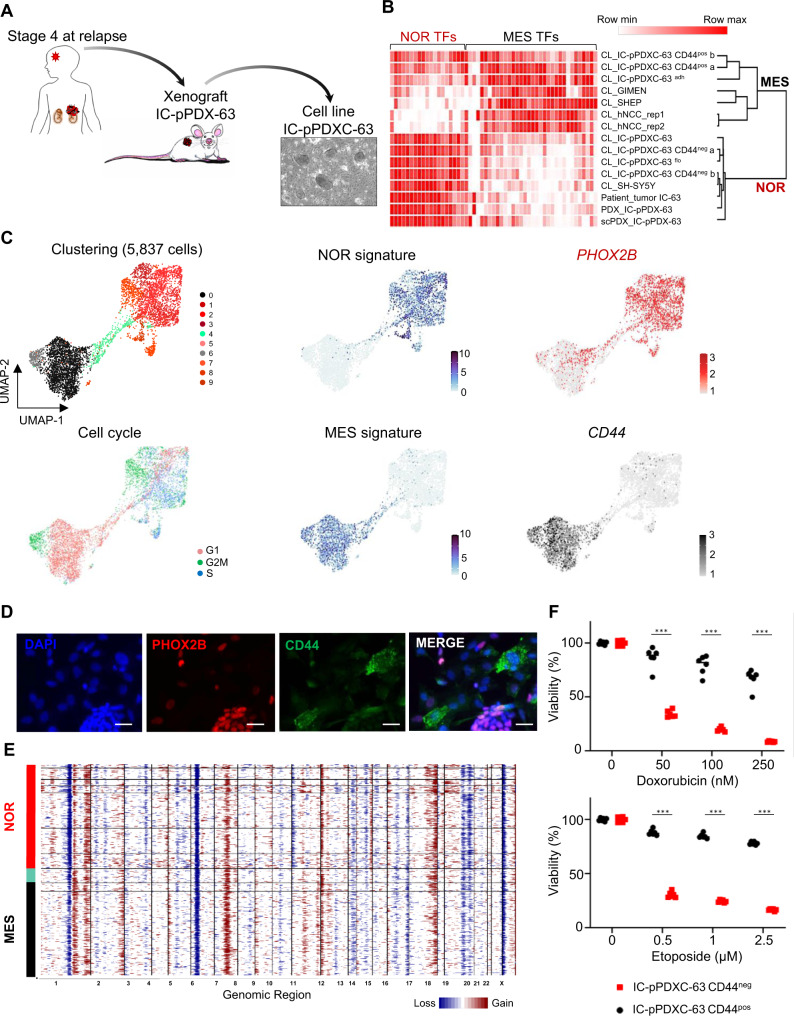
IC-pPDXC-63 is an original model of phenotypic heterogeneity including noradrenergic and mesenchymal tumor identities. NOR noradrenergic, MES mesenchymal. **A** Ex vivo culture of the noradrenergic IC-pPDX-63 neuroblastoma model established from a brain metastasis of a stage 4 case at relapse resulted in the IC-pPDXC-63 cell line that includes floating neurospheres and adherent cells as observed by contrast phase microscopy. **B** Heatmap and unsupervised clustering of samples (cell lines, patient tumors and PDXs) using the expression of transcription factors (TFs) of the noradrenergic and mesenchymal identities^17,18^ on bulk RNAseq data. The transcriptomic profile of the IC-pPDX-63 model (bulk RNAseq of the PDX tumor (PDX) or from its single-cell (scPDX)) and its derived-cell line (CL_IC-pPDXC-63) are noradrenergic and highly similar to that of the matched patient tumor (IC-63) from which it has been generated. Two replicates of CD44^pos^ and CD44^neg^ cell sorts of the cell line are included (a and b). CL cell line, flo floating neurospheres, adh adherent cells. hNCC human neural crest cells. Source data are provided as a Source Data file. **C** Single-cell transcriptomic analyses of the IC-pPDXC-63 cell line by Seurat showing clustering at resolution 0.8, cell cycle phases, and the noradrenergic and mesenchymal identities highlighted by noradrenergic and mesenchymal transcription factor signatures^17,18^ and *PHOX2B* and *CD44* expression, respectively, plus a bridge in-between. **D** Immunofluorescence shows the specific expression of the PHOX2B and CD44 markers by neurospheres and adherent cells, respectively (scale bar = 20 µm, representative of 3 independent experiments). **E** Inferred genomic profile of the IC-pPDXC-63 cell line obtained with InferCNV on single-cell data. **F** Mesenchymal (IC-pPDXC-63 CD44^pos^) cells are more resistant to chemotherapy than noradrenergic (IC-pPDXC-63 CD44^neg^) cells. Cell viability was measured with resazurin assay after 72 h of chemotherapy treatments (Doxorubicin 50, 100, 250 nM and Etoposide 0.5, 1, 2.5 µM, mean ± sd; *n* = 6 replicates). *P*-values were determined via two-tailed unpaired Welch’s *t*-test (***p < 0.000001). Source data are provided as a Source Data file.

In order to demonstrate that this heterogeneity in tumor cell identity is not exclusively observed in the SK-N-SH sample, we generated new cell lines from a series of 15 neuroblastoma PDX models. Cells from five models could be maintained in culture for several months and frozen. Three derived-cell lines had a pure noradrenergic phenotype. Interestingly, two others (IC-pPDXC-63 and IC-pPDXC-109) were able to grow in vitro as a bi-phenotypic culture, with both adherent cells and neurospheres (Fig. 2A and Supplementary Fig. 1A). Bulk RNA-seq analysis confirmed that the PDX models (IC-pPDX-63 and IC-pPDX-109) and their derived-cell line exhibited a transcriptomic profile highly similar to the one of the patient tumor (Fig. 2B and Supplementary Fig. 1B). As shown by single-cell analysis, two main clusters of noradrenergic cells and mesenchymal tumor cells, connected by a bridge population, were observed in the IC-pPDXC-63 cell line (Fig. 2C). As expected, noradrenergic cells expressed *PHOX2B*, whereas the mesenchymal population expressed the cell surface *CD44* marker. The clustering separating noradrenergic and mesenchymal tumor cells was not biased by the cell cycle (Fig. 2C). These two tumor identities were also identified in the IC-pPDXC-109 cell line (Supplementary Fig. S1C). Immunofluorescence on IC-pPDXC-63 confirmed that CD44 and PHOX2B were specifically expressed by adherent cells and neurospheres, respectively (Fig. 2D). Bulk RNA-seq analysis endorsed that CD44^pos^ FACS-sorted cells and adherent cells have a transcriptomic profile close to the SH-EP cells or other mesenchymal cell lines, whereas CD44^neg^ FACS-sorted cells and neurospheres clustered with the noradrenergic SH-SY5Y cells (Fig. 2B). Of note, inferred genomic alterations were highly similar in noradrenergic and mesenchymal tumor cells in both IC-pPDXC-63 and IC-pPDXC-109 cell lines (Fig. 2E and Supplementary Fig. 1D). Consistently with our observations in the SK-N-SH cell line, we documented that the mesenchymal population of the IC-pPDXC-63 cell line exhibited a higher chemo-resistance compared to the noradrenergic one (Fig. 2F).

### Tumor cell plasticity from noradrenergic towards mesenchymal state reveals the reprogramming potential of a subset of neuroblastoma cells

**Fig. 3:**
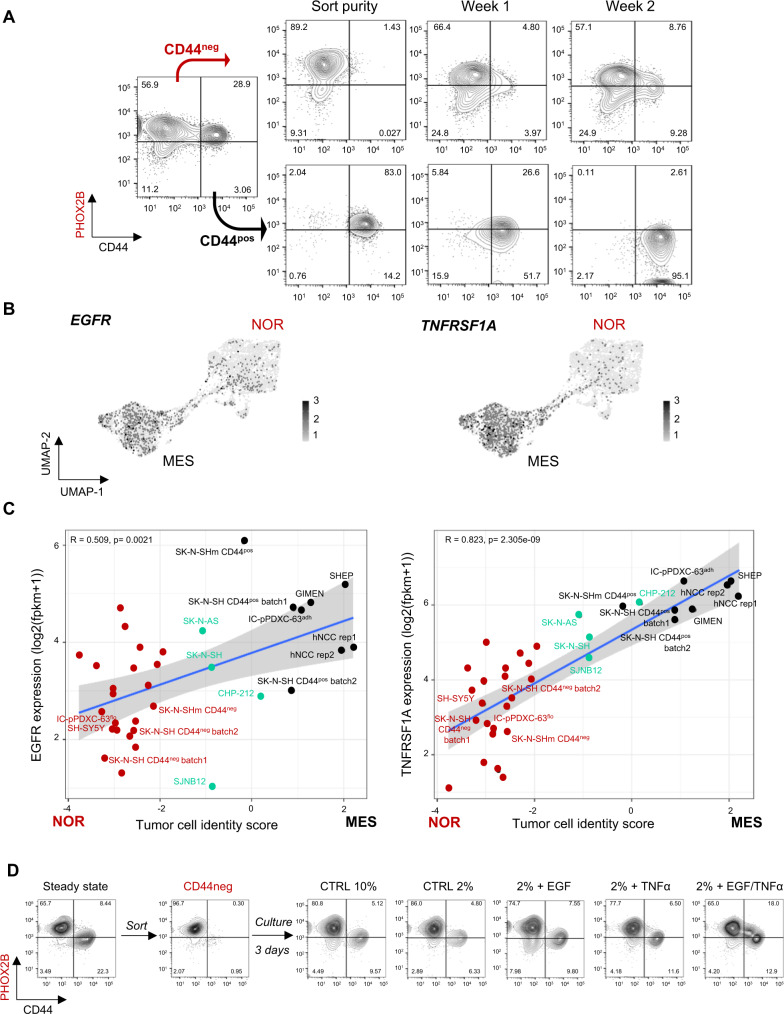
Noradrenergic to mesenchymal plasticity can be induced by extrinsic factors including EGF and TNFα. NOR noradrenergic, MES mesenchymal. **A** Noradrenergic to mesenchymal shift of identity of the IC-pPDXC-63 cell line. Mesenchymal/CD44^pos^ and noradrenergic/CD44^neg^ cells were FACS sorted and cultured for two weeks. Only the CD44^neg^ sorted population reconstituted a heterogeneous cell population after several days in culture. Representative FACS analyses of PHOX2B and CD44 staining gated on live cells after doublet exclusion are shown. **B** Umap plot of *EGFR* and *TNFRSF1A* gene expression in scRNAseq of IC-pPDXC-63. **C** Scatterplot showing the correlation of *EGFR* and *TNFRSF1A* gene expression by RNAseq in each cell line with the tumor cell identity score (score MES - score NOR). Noradrenergic and mesenchymal scores correspond to the mean expression of transcription factors that define each identity^17,18^. Simple linear regression line is shown, and gray cloud represents the 95% two-tailed confidence interval of the slope. The measure of linear association is given by Pearson’s product moment correlation production (r) with its p-value (TNFRSF1A: *t* = 8.1978, df = 32, 95% CI for r [0.6719094, 0.9083932]; EGFR: *t* = 3.3464, df = 32, 95% CI for r [0.2065378, 0.7228518]). Color code: red = noradrenergic, black = mesenchymal, green = intermediate cell lines. Source data are provided as a Source Data file. **D** Representative FACS analysis using PHOX2B and CD44 marker expression gated on live cells after doublet exclusion to follow the noradrenergic to mesenchymal shift of identity of the SK-N-SHm cell line. CD44^neg^ cells were culture in control conditions (CTRL, 2% FBS) or with EGF and TNFα growth factors (20 nM each) for 48 h.

To explore the plasticity of the various tumor cell populations, SK-N-SH and IC-pPDXC-63 noradrenergic/CD44^neg^ and mesenchymal/CD44^pos^ cells were FACS-sorted and cultured separately (Supplementary Fig. 2A, B). Their plasticity potential was established based on the phenotype, followed by FACS analysis done weekly for 2 weeks. This experiment was performed on IC-pPDXC-63 and on two batches of SK-N-SH and consistently demonstrated in both models that only the noradrenergic/CD44^neg^ cells were able to give rise to a heterogeneous population comprising both CD44^neg^ and CD44^pos^ cells. In contrast, the CD44^pos^ fraction that contained <0.5% of contaminant noradrenergic cells was unable to generate heterogeneity (Fig. 3A and Supplementary Fig. 2B). From the noradrenergic/CD44^neg^ cells of the parental SK-N-SH cell line batch 2, we derived the SK-N-SHm cell line, comprising both CD44^neg^ and CD44^pos^ cells that were further characterized by single-cell RNAseq, IF, FACS and bulk RNAseq (Supplementary Fig. 3A–D). These results demonstrate that the noradrenergic to mesenchymal plasticity potential is maintained across several cell generations (Supplementary Fig. 2C, D). Of note, when inferring the genetic alterations of the SK-N-SH batch 1 and the SK-N-SHm from batch 2, we observed differences on chromosomes 1 and 2 (Supplementary Fig. 3E). These observations are consistent with the previous description of different genetic subclones in the parental SK-N-SH cell line^24,30^. However, identical copy number profiles were shared by both noradrenergic and mesenchymal cells (one cluster out of 2), showing that the presence of these CNVs was not directly related to cell identity and plasticity.

Next, we sought to identify some extrinsic factors that could influence the noradrenergic to mesenchymal transition (NMT). We therefore looked at growth factor receptor expression in our cellular models of plasticity. We found that *EGFR* and *TNFRSF1A*, receptors of the EGF and TNFα cell signaling pathways, respectively, were expressed in the bridge and mesenchymal cells from IC-pPDXC-63 (Fig. 3B), in the mesenchymal cells of SK-N-SH and more generally associated to mesenchymal tumor identity using a set of 25 neuroblastoma cell lines (Fig. 3C). As the factors inducing NMT are probably present in the serum, we reduced its concentration from 10 to 2% and observed a decrease in the number of SK-N-SH CD44^pos^ cells spontaneously obtained after 3 days of culture of CD44^neg^ cells. The treatment of SK-N-SH noradrenergic/CD44^neg^ cells for 3 days in 2% FBS complemented with EGF and TNFα strongly promoted the NMT, decreasing the PHOX2B^pos^/CD44^neg^ population and increasing the total percentage of CD44^pos^ cells up to 30.9% in comparison to 11.13% in the control condition. Of note, the combination of EGF and TNFα allowed to clearly observe an intermediate phenotype between the two cell populations detected in the control condition (Fig. 3D).

These results therefore highlight the plasticity potential orientated from a noradrenergic to a mesenchymal state of two distinct models that could be stimulated by extrinsic growth factors including EGF and TNFα.

### Phenotypic plasticity is associated with epigenetic reprogramming

**Fig. 4:**
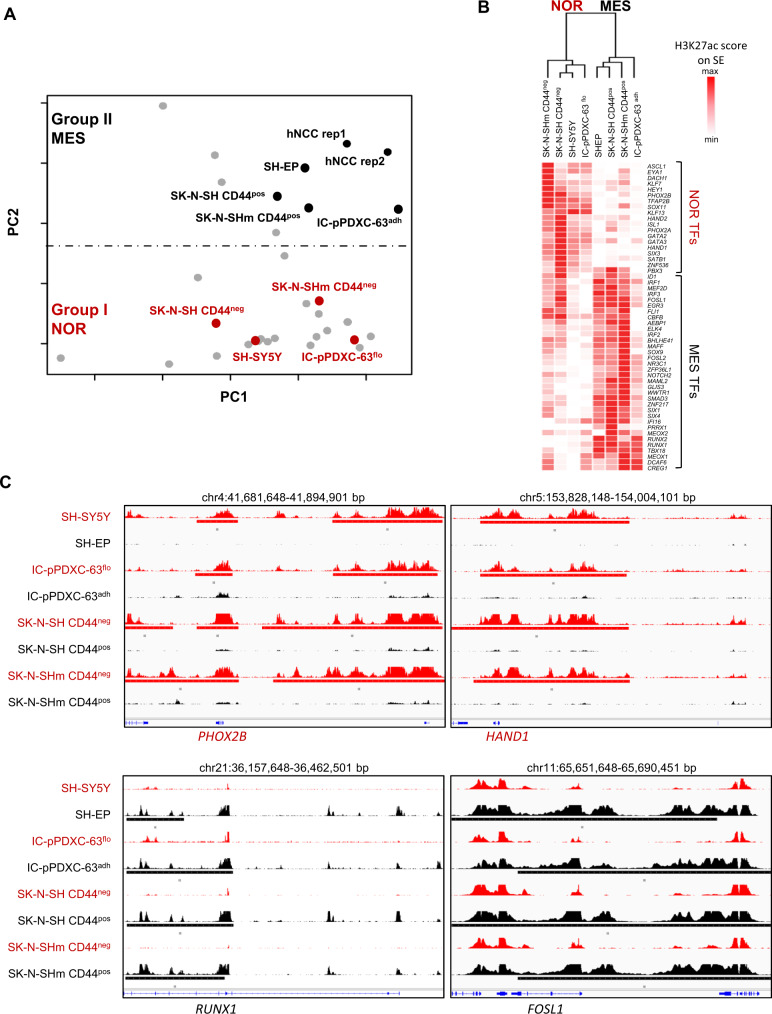
The noradrenergic to mesenchymal plasticity is associated with an epigenetic reprogramming. NOR noradrenergic, MES mesenchymal. **A** Principal component (PC) analysis based on neuroblastoma and hNCC super-enhancer log scores^17^ that discriminates the two neuroblastoma cell groups I (noradrenergic) and II (NCC-like/mesenchymal) and in which were added the floating and adherent cells of the IC-pPDXC-63 and CD44^neg^ and CD44^pos^ sorted cells of the SK-N-SH cell line. **B** Heatmap showing the H3K27ac signal on the super-enhancer regions of the transcription factors (TFs) of the noradrenergic and mesenchymal identities^17,18^ in the floating and adherent cells of the IC-pPDXC-63 cell line and in the CD44^pos^ and CD44^neg^ sorted cells of the SK-N-SH cell lines, and the SH-SY5Y and SH-EP control cell lines. For the TFs associated with several super-enhancers, the signal was summarized as described in the “Methods” section to have one value per TF. The unsupervised hierarchical clustering based on H3K27ac signals discriminated noradrenergic and mesenchymal cell identity. Source data are provided as a Source Data file. **C** IGV tracks of ChIP-seq profiles (scale 0-100) for H3K27ac at *PHOX2B, HAND1, RUNX1*, and *FOSL1* super-enhancers in the SH-SY5Y, SH-EP, the floating and adherent IC-pPDXC-63 cells and the CD44^pos^ and CD44^neg^ sorted SK-N-SH and SK-N-SHm cells.

To deeper characterize the epigenetic reprogramming contribution to cell plasticity, we next defined the super-enhancer landscape of the different models (the two batches of SK-N-SH and IC-pPDXC-63 cell lines) by ChIP-seq analyses for the H3K27ac mark. We added these samples in the principal component analysis (PCA) of neuroblastoma cell lines and hNCC lines based on their super-enhancer log scores^17^ (Fig. 4A). Their mesenchymal counterparts, i.e., SK-N-SH CD44^pos^ FACS-sorted and adherent IC-pPDXC-63 cells, showed an epigenetic profile close to the group II of mesenchymal identity. Consistently, their noradrenergic counterparts, the SK-N-SH CD44^neg^ FACS-sorted and floating IC-pPDXC-63 cells were part of the noradrenergic cell line group I (Fig. 4A). For the mesenchymal cells, a low H3K27ac signal could be quantified for the super-enhancer regions of the noradrenergic CRC such as *GATA3*, *PHOX2B* and *HAND1*. On the other hand, a high H3K27ac signal could be observed on *RUNX1*, *FOSL1*, *NR3C1,* and *TBX18*, previously described as NCC-like/mesenchymal transcription factors^17^ (Fig. 4B, C). Altogether, these results indicate that the spontaneous shift from a noradrenergic to a mesenchymal identity observed for SK-N-SH and IC-pPDXC-63 cells is associated with epigenetic reprogramming.

### Mesenchymal neuroblastoma cells are reversely reprogrammed to a noradrenergic phenotype in vivo

**Fig. 5:**
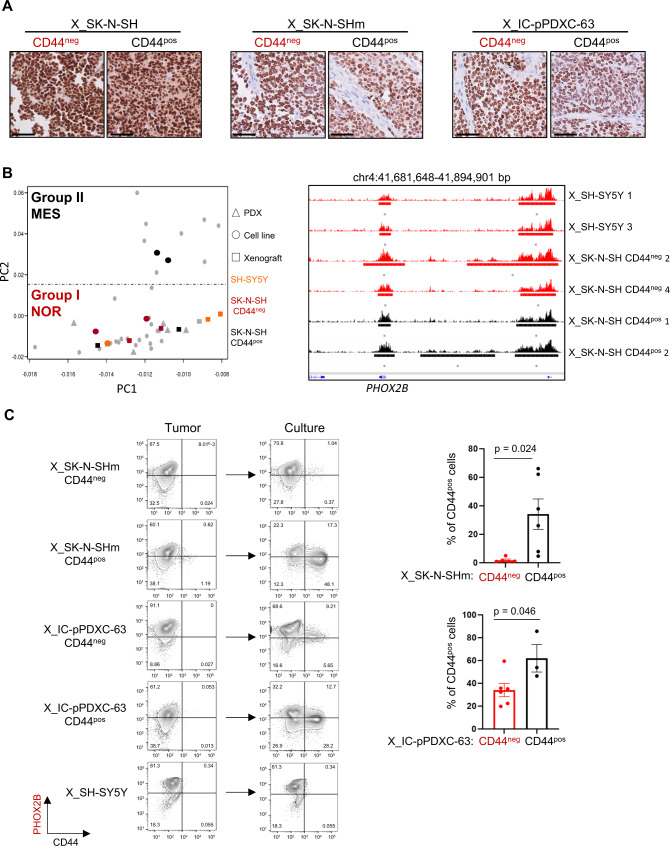
Reversible mesenchymal to noradrenergic shift in the SK-N-SH and IC-pPDXC-63 models. NOR noradrenergic, MES mesenchymal, X xenograft. **A** PHOX2B immunohistochemistry of one representative mouse xenograft per group (obtained from CD44^neg^ or CD44^pos^ sorted cells of SK-N-SH, SK-N-SHm and IC-pPDXC-63 cell lines). Scale bar = 50 µm. Similar results were obtained for all analyzed xenografts. **B** Left: PCA based on super-enhancer log scores^17^ as in Fig. 4A in which were added the xenografts of the SH-SY5Y and the xenografts of the CD44^pos^ or CD44^neg^ sorted cells of the SK-N-SH cell line. Right: tracks of ChIP-seq profiles (scale 0-100) for H3K27ac at *PHOX2B* super-enhancer in the xenografts of the SH-SY5Y and SK-N-SH cell populations sorted according to CD44 expression. **C** Left panels: FACS analyses using PHOX2B and CD44 marker expression gated on live cells after doublet exclusion comparing the profile of cells from the xenograft tumor at ethical size and the same cells cultivated until they reached confluence. Conditions are SK-N-SHm and IC-pPDXC-63 cells, CD44^pos^ or CD44^neg^ FACS sorted before engraftment in mice, and SH-SY5Y. Right panels: Quantification of CD44^pos^ cells by FACS after culture, mean ± sd; *n* = 6 replicates except for X_IC-pPDXC-63 CD44^pos^ *n* = 3, unpaired two-tailed *t*-test, *p < 0.05, each dot corresponds to one xenograft. Source data are provided as a Source Data file.

We next investigated the behaviors of noradrenergic and mesenchymal cell populations in vivo. The noradrenergic/CD44^neg^ and mesenchymal/CD44^pos^ FACS-sorted cell populations from the SK-N-SH and IC-pPDXC-63 cell lines were injected subcutaneously into mice. Tumors developed in all cases, indicating that the different states displayed tumorigenic potential in vivo (Supplementary Fig. 4A). Unexpectedly, as revealed by IHC analysis, PHOX2B expression was observed in most tumor cells from the whole set of xenografts, even those obtained after engraftment of mesenchymal populations (Fig. 5A). Bulk RNA-seq experiments confirmed that all tumors highly expressed the transcription factors of the noradrenergic CRC and exhibited a noradrenergic transcriptomic profile (Supplementary Fig. 4B). Noradrenergic and neuroendocrine markers such as *DBH, NET/SLC6A2, CHGA* and *CHGB* were similarly expressed in xenografts obtained from both CD44^pos^ and CD44^neg^ cell populations of the SK-N-SH and IC-pPDXC-63 cell lines (Supplementary Fig. 4B). Finally, to fully demonstrate the in vivo reprogramming of mesenchymal cells towards a noradrenergic identity, we determined the super-enhancer profiles of the xenografts of SK-N-SH CD44^pos^ and CD44^neg^ FACS-sorted cells. The PCA on H3K27ac signals clearly showed that all these tumors were part of the noradrenergic group I (Fig. 5B). Super-enhancers marked *PHOX2B* in xenografts of SH-SY5Y and SK-N-SH CD44^neg^ but also in xenografts of SK-N-SH CD44^pos^ cells (Fig. 5B). In vivo, the mesenchymal cells therefore shifted back towards a noradrenergic identity indicating that the mouse microenvironment provided strong cues inducing a global epigenetic reprogramming.

When tumors reached ethical size, mice were sacrificed. Tumors were dissociated, cells were grown in vitro and cell identity was followed by CD44 FACS analysis. Interestingly, the ability of cells to shift from a noradrenergic to mesenchymal identity in vitro was again observed for cells from both models but not from SH-SY5Y xenografts (Fig. 5C). Of note, cells from xenografts initially generated from mesenchymal cells were more efficient to generate mesenchymal cells than cells from xenografts generated from noradrenergic cells, suggesting some form of memory of their initial identity (Fig. 5C).

Altogether, our analyses highlighted the plasticity properties of neuroblastoma cells using two different models, the SK-N-SH and IC-pPDXC-63 cell lines. This plasticity was associated with a reprogramming potential from a mesenchymal state to a noradrenergic state following engraftment in the mouse. We demonstrate that this plasticity is reversible since a reprogramming from a noradrenergic to a mesenchymal state was observed when cells were cultured back in vitro.

### Single-cell transcriptomic analyses reveal intra-tumor noradrenergic heterogeneity and noradrenergic cells with mesenchymal features in patients

**Fig. 6:**
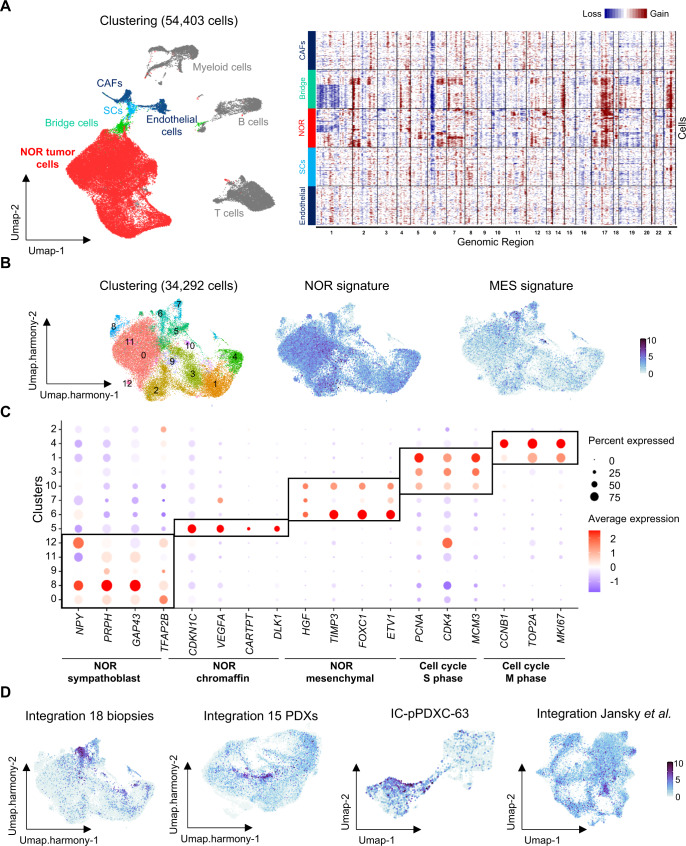
Single-cell transcriptomic reveals a noradrenergic population with mesenchymal features in neuroblastoma patients. NOR noradrenergic, MES mesenchymal. **A** (Left) Umap of the 54,403 cells from 18 biopsies integrated with Seurat. (Right) InferCNV analysis on 600 randomly selected cells from four clusters. Color code: gray = immune cells, dark blue = mesenchymal cells (endothelial cells and cancer-associated fibroblasts CAFs), light blue = Schwann cells (SCs), red = NOR tumor cells, green = bridge cells. **B** Umap of the clustering of the 34,292 tumor cells integrated with Harmony, extracted from the 18 biopsies. Plots of noradrenergic and mesenchymal transcription factor signatures^17,18^. **C** Dot plot graph illustrating cluster-specific gene expression. Three main tumor cell identities could be defined: noradrenergic tumor cells with either sympathoblast, chromaffin or mesenchymal features. **D** Plot of the signature identifying the noradrenergic cells with mesenchymal features composed of *HGF*, *TIMP3*, *FOXC1,* and *ETV1* in the integration of single-cell RNAseq data of biopsies and PDXs, in the IC-pPDXC-63 cell line and in an independent cohort of 14 neuroblastoma cases already published^13^.

We next explored neuroblastoma intra-tumor heterogeneity using single-cell transcriptomic analyses with the 10X Genomics technology on a series of 18 patient biopsies and our series of 15 PDX models. The analyzed samples have been obtained at diagnosis, progression or relapse (Supplementary Tables 1 and 2). The integration of the 18 biopsies (*n* = 54,403 cells, Supplementary Table 3) highlighted several cell populations of the microenvironment as previously described^31^ with various clusters of immune cells (myeloid, B and T cells) and mesenchymal cells corresponding to endothelial cells, cancer-associated fibroblasts (CAFs) and Schwann cells. We identified tumor cells of noradrenergic identity and a cluster of bridge cells with a transcriptional signature in between noradrenergic tumor cells and Schwann cells (Fig. 6A and Supplementary Fig. 5A). Importantly, this bridge population was clearly of tumor origin as it shared the same emblematic genetic alterations of neuroblastoma^1^ (i.e. 17q gain or 1p loss) as noradrenergic cells whereas no such alterations were inferred from endothelial cells, Schwann cells and CAFs clusters (Fig. 6A). Next, we extracted all tumor cells exhibiting copy number variations as detected by InferCNV and integrated them with Harmony^32^ (*n* = 34,292 cells) (Fig. 6B and Supplementary Fig. 5B). Most clusters were shared by all cases; yet, some clusters (6, 7, 8, 11 and 12) included cells from a subset of patients only (Supplementary Fig. 5C). Interestingly, several clusters including cluster 6 were highlighted by both the noradrenergic and mesenchymal signatures (Fig. 6B). Marker genes that define cell clusters were then identified after differential expression analysis (Supplementary Data 1). Cluster 6 mainly (but also clusters 7 and 10) could be defined by genes related to mesenchymal features and plasticity through EMT such as *FOXC1*^33^*, TIMP3*^34^, *ETV1*^35^, and *HGF* and corresponded to the bridge population seen at the interface between noradrenergic tumor cells and normal Schwann cells in Fig. 6A (Fig. 6C, Supplementary Fig. 5D and Supplementary Data 1). Several other populations were identified according to the up-regulated expression of specific genes: cycling cells, cells expressing chromaffin markers such as *CARTPT*^36,37^, *DLK1*^38,39^, and *CDKN1C* (cluster 5) and cells with a sympathoblast-like identity expressing genes such as *TFAP2B*^40^, *PRPH*^41^, *GAP43,* and *NPY*^42^ (clusters 0, 8, 9, 11, and 12) (Fig. 6C and Supplementary Data 1). Published signatures from human adrenal development^13^ were used to corroborate our cluster identification (Supplementary Fig. 5E). We noticed that tumor cells with either sympathoblast or chromaffin-like identities co-existed in the same tumors (Supplementary Fig. 5C).

We next investigated our cohort of 15 PDXs. The relevance of these models was assessed by an unsupervised hierarchical clustering which revealed strong similarities between transcriptomes of matched patient tumors and PDXs (Supplementary Fig. 6). Moreover, InferCNV profiles calculated from scRNAseq data were fully consistent with CNVs calculated from available WES (Supplementary Fig. 7A). In a further step, integration of all human cells (*n* = 58,120 cells) from the 15 PDXs was generated (Supplementary Fig. 7B–D, Supplementary Data 2). The three main tumor identities defined in the 18 biopsies as noradrenergic with sympathoblast (clusters 1, 6 and 8), or chromaffin (clusters 4 and 9) or mesenchymal (cluster 7) features could be also observed in the cohort of the 15 PDXs (Supplementary Fig. 8A, B). We next applied the NOR-mesenchymal signature (the four genes *TIMP3*, *FOXC1*, *ETV1,* and *HGF*) allowing to specifically distinguish the noradrenergic cells with mesenchymal features in the 18 patient biopsies on our PDX models. As expected, this signature highlighted cluster 7, defined as the noradrenergic tumor cells with mesenchymal features in the integration of the 15 PDXs (Fig. 6D). Of note, this cluster contains cells from IC-pPDX-63 and IC-pPDX-109 (Supplementary Figs. 7C and 8C), the two models that gave rise to mesenchymal tumor cells in vitro. This signature was also expressed by the bridge and the mesenchymal tumor cells from the IC-pPDXC-63 cell line (Fig. 6D). Finally, the three tumor cell identities, NOR-chromaffin, NOR-sympathoblast or NOR-mesenchymal, did not seem to be influenced by the diagnosis or relapse status of the corresponding patients, nor by their genomic alterations (Supplementary Figs. 8D, E and 9).

Finally, to extend our results to other datasets, we reanalyzed the series of 14 cases studied by snRNAseq by Jansky et al.^13^, extracted the noradrenergic tumor cells and applied our signatures. Interestingly, the NOR-mesenchymal signature highlighted a specific cluster of cells (cluster 10) that was mostly composed by cells from one case (Supplementary Fig. 10 and Fig. 6D).

Altogether, these results on both neuroblastoma patient biopsies and PDX models characterize neuroblastoma intra-tumor heterogeneity with the identification of cells with sympathoblast or chromaffin features and, in a subset of patients, cells with a noradrenergic identity concomitantly expressing mesenchymal features, reminiscent of the cellular models exhibiting plasticity between a noradrenergic and a mesenchymal identity.

## Discussion

Our present work further reports intra-tumor heterogeneity in neuroblastoma cell lines, PDXs and patient tumors and documents several features of cell plasticity. First, we describe a reversible spontaneous, non-genetic, reprogramming potential between a noradrenergic and a mesenchymal identity for the SK-N-SH and IC-pPDXC-63 neuroblastoma cell lines. Single-cell transcriptomic analyses performed on these two cellular models identified CD44 as a surface marker specific of the mesenchymal identity, further allowing the use of this marker to sort each population and analyze their respective proportions by FACS in vitro. This marker is particularly useful as the CD133 cell surface protein, previously suggested as a marker discriminating noradrenergic and mesenchymal cells^18^, has been shown to be downregulated upon in vitro culture with serum-containing media. In both models, neuroblastoma plasticity can be orientated from a noradrenergic towards a mesenchymal identity in vitro, especially in response to EGF and TNFα factors. These pathways have previously been linked to a change of tumor identity in the noradrenergic SH-SY5Y cell line^43^. Our data also document that the mesenchymal to noradrenergic shift was favored by the in vivo microenvironment.

Second, we demonstrate that the spontaneous plasticity between the noradrenergic and mesenchymal states relies on a profound epigenetic reprogramming, as revealed by the analysis of the super-enhancer landscape, and the expression of the transcription factors of the CRCs previously characterized for each cell identity^17,18^. Interestingly, we could document that the reprogramming potential associated with plasticity is maintained through several generations in the SK-N-SH cell line. Finally, our experiments suggest a memory of cell identity as noradrenergic tumor cells obtained from grafts of CD44^pos^ cells in mice more efficiently undergo in vitro mesenchymal transition, as compared to noradrenergic tumor cells obtained from grafts of CD44^neg^ cells. Our study draws in neuroblastoma a parallel with the well-studied epithelial-mesenchymal transition (EMT), a crucial process for embryogenesis and wound healing but largely hijacked in carcinomas^44–46^. The mesenchymal features have been associated with tumor initiation, invasion, metastasis and resistance to therapy. Fifty years after its pioneer observation, initially thought to be a binary process, EMT now resides in a large spectrum of intermediate states between a fully epithelial and mesenchymal phenotype^46,47^. The ability of cells to adopt mixed identities and/or interconvert from one to another define the notion of epithelial-mesenchymal plasticity, that can even be encountered in non-epithelial cancers such as melanoma^48^ and sarcoma^49^. In several tumor types, cellular and mouse models have been used to demonstrate that intermediate states can be associated with distinct molecular and functional properties^50,51^. However, while epithelial characteristics are clearly defined, no consensus has been reached today in the EMT community to define the mesenchymal state with universal molecular and cellular markers^46^. Mesenchymal-epithelial transition (MET), the reversion of EMT, also occurs during development and tumor metastasis but is even less studied and understood than EMT. Thus, experimental strategies combined with computational approaches are still required to understand the functional roles of various populations and cell states, particularly in patients^52^. Using neuroblastoma cellular models, we now demonstrate that some noradrenergic cells are able to shift towards a state with mesenchymal features, implying a NMT^26^, but we also document the reverse process, MNT. As for EMT/MET, external signals from the microenvironment as well as intrinsic molecular events (signaling pathways, transcription factors, epigenetic actors, chromatin remodeling) remain to be identified to deeply characterize NMT/MNT and their role in metastasis and resistance to treatment, two hallmarks of high-risk neuroblastoma.

So far, the in vitro inactivation of the noradrenergic CRC transcription factors has been mostly obtained in exome-wide CRISPR-Cas9 screens or in short-term interference experiments unveiling genes essential for survival and/or growth but without any demonstration of cell identity shift^20^. Only the knock-out of the *ARID1A* gene in clones of the NGP cell line has been shown to be associated with a shift from a noradrenergic to a mesenchymal identity^53^. Manipulating transcription factors of the mesenchymal CRC provided interesting results. Indeed, the overexpression of PRRX1^18^ or NOTCH3 intracellular domain^25^ in noradrenergic cells was sufficient to push cells towards a mesenchymal identity. Xenografts of SH-SY5Y cells overexpressing NOTCH3 intracellular domain exhibited a mesenchymal identity, in contrary to our experiments of engraftments of CD44^pos^ cells forming systematically noradrenergic tumors. The permanent production of intra-cellular NOTCH3 by the SH-SY5Y cells likely activates an endogenous feed-forward loop between NOTCH receptors and ligands, allowing the maintenance of a mesenchymal state in vivo. The complete description of the signals driving the activation of the NOTCH pathway or regulating PRRX1 expression in noradrenergic cells is further needed to evaluate a possible role of the NMT in chemoresistance processes.

Since their characterization in vitro, mesenchymal tumor cells have been intensely pursued in neuroblastoma patients. Immunohistochemistry applied to diagnosis/relapse cases suggested an enrichment of mesenchymal cells after treatments of patients^18^, however without the use of any specific tumor mesenchymal marker it remains difficult to ascertain their tumor identity. Bulk transcriptomic and/or epigenetic analyses identified few cases enriched in mesenchymal signature scores^23^, but with the limitation of analyzing a mix of tumor cells and normal cells of the microenvironment. To bypass those difficulties linked to bulk analyses, several teams have now applied single-cell approaches to dissect intra-tumor heterogeneity in neuroblastoma^12–16^. Such approaches, providing a more global view of each cell transcriptome and identity allow the characterization of several thousands of cells that yet may not be fully representative of a whole tumor. In these recently published studies including ours, no distinct cluster of ‘pure’ mesenchymal tumor cells as observed in vitro has been described so far in patient tumors, nor PRRX1-positive tumor cells as previously proposed^18^. A transitional state between the noradrenergic and mesenchymal states has been proposed recently using pseudotime trajectory analyses on scRNA-seq data obtained from a series of neuroblastoma, ganglioneuroblastoma and ganglioneuroma^54^. Yet, these transitional cells did not appear in a path or in a cluster in between noradrenergic and mesenchymal cells. Moreover, no genomic alterations common to both states were observed in favor of cell plasticity. In our patient tumor dataset, we identified a cluster of noradrenergic tumor cells forming a bridge towards a cluster of normal Schwann and mesenchymal cells and composed of cells of three tumors at diagnosis and one case at relapse. These cells are unambiguously tumor cells exhibiting copy number alterations that co-express markers of a noradrenergic identity and markers of mesenchymal features. Defining a signature of those noradrenergic tumor cells with mesenchymal features allowed us to highlight a specific cluster in our integration of 15 PDXs and in an independent published dataset of patient biopsies^13^. Interestingly, the cluster in PDX was composed of three cases including the IC-pPDX-63 and IC-pPDX-109 models, the two models from which we could obtain bi-phenotypic cell lines with noradrenergic and mesenchymal tumor cells in vitro. The convergence of these observations, based on orthogonal approaches (i.e. analyses of experimental models and of patient’s tumors), strongly suggests that cells with such a signature are the ones exhibiting a reprogramming potential towards a full mesenchymal identity in vitro. The observation that CD44^pos^-FACS sorted/mesenchymal cells from our heterogeneous and plastic models give rise to full noradrenergic tumors in vivo underlies the powerful pressure towards a noradrenergic state for tumor formation. Altogether, these data may suggest that, in vivo, the mesenchymal identity could represent a very transient state, very challenging to capture in patient biopsies performed during disease progression with the available technologies. Nevertheless, this rare population of cells could play a major role at the early steps of the metastatic process and/or under therapy pressure. Given their plasticity potential, those cells could be at the origin of metastases or resistant cells to treatment. Yet, our present results suggest that cells in an intermediate noradrenergic/mesenchymal state are detected in a subset of patients. Interestingly, the definition of a NOR-mesenchymal signature (*FOXC1, TIMP3*, *ETV1*, and *HGF)* allowed to identify this population of noradrenergic cells with mesenchymal features in patients, PDXs and in cellular models. This signature does not include the *MYCN*, *NEUROD6*, *EZH2* and *SOX11* genes that define the transitional state in the study by Yuan et al.^54^. Our data rather highlight upregulation of *EZH2* and *SOX11* in clusters of cycling cells and *MYCN* in cluster 7 associated with a 2p amplification, whereas *NEUROD6* is expressed only in rare cells. Further single cell analyses combining cell tag barcoding, spatial and/or deeper sequencing should allow the precise molecular, cellular and functional characterization of those peculiar cells.

Interactions between tumor cells and the microenvironment may influence the tumor cell phenotype and play a role in tumor progression, as previously demonstrated for tumor-associated inflammatory cells^55,56^. Future studies should decipher the cues of the microenvironment and their associated pathways that converge to regulate cell plasticity during tumor progression. Recently, olfactomedin-1 has been identified as an environmental signal produced by chick embryonic sympathetic ganglia and able to trigger NMT and dissemination of neuroblastoma cells in an avian model^57^. Furthermore, it has been suggested that therapies against neuroblastoma may impact cell identity. Indeed, previous analyses of neuroblastoma cells selected to be resistant to cisplatin^58^ or ALK inhibitors^59^ in vitro have reported that noradrenergic cells may acquire mesenchymal properties.

The cell of origin in neuroblastoma has been for long a matter of debate^60–62^. Some models argue that this tumor arises from the transformation of NCCs while other models suggest that they develop from more engaged sympatho-adrenal progenitors, able to generate both sympathetic neurons and neuroendocrine cells of the adrenal. Recently, this hierarchical dogma of normal differentiation has been questioned with the identification of a population of Schwann cell precursors (SCPs) as the main reservoir of adrenal chromaffin cells in the mouse^63^. In several recent papers which analyzed human fetal adrenal gland development at the single-cell level in addition to adrenal neuroblastoma tumors, authors concluded that neuroblastoma cells mainly resemble normal fetal adrenal neuroblasts or embryonic sympathoblasts^13,15,64^. Only Dong et al. concluded that malignant cells had a predominant chromaffin-cell-like phenotype^14,36,65,66^. Our data integrations of 18 biopsies and 15 PDX cases revealed two distinct tumor cell populations linked to normal sympathetic development: one expressing markers of chromaffin cells and one expressing markers of sympathoblasts. Interestingly, these two populations seemed to co-exist in some tumor cases. Of note, SCP markers such as *SOX10, S100B, PLP1,* and *ERBB3*^63^ were not detected in tumor cells. Nevertheless, it remains difficult to infer from such approaches which cell type is the one targeted by neoplastic transformation. Indeed, it cannot be excluded that transitions may occur between chromaffin cells and sympathoblasts during development and/or that markers of a specific precursor may be lost during cell transformation.

Altogether, our data obtained on several cellular models and tumor patients demonstrate that a subset of neuroblastoma cells exhibits a reprogramming potential between a noradrenergic and a mesenchymal identity and that both intrinsic properties and exogenous signals of the microenvironment dictate these identities. A better understanding of the molecular factors that control phenotypic plasticity using either more in-depth sequencing, and/or epigenetic information at single-cell level and larger series of patients will represent a key step in the design of more effective therapies that aim at improving the outcome of neuroblastoma patients with high-risk disease.

## Methods

The study complies with all relevant ethical regulations and was approved by the Institut Curie’s Institutional Review Board.

### Experimental models

#### Neuroblastoma cell lines

The two batches of SK-N-SH (Cat# HTB-11, RRID:CVCL_0531) and SH-SY5Y (Cat# CRL-2266, RRID:CVCL_0019) cell lines have been obtained from the ATCC. The IC-pPDXC-63 cell line was derived from the IC-pPDX-63 model, and the IC-pPDXC-109 from IC-pPDX-109. Cell line authentication was done by STR profiling with PowerPlex® 16 HS System from Promega and Cytoscan HD array (Affymetrix).

All cells were grown at 37 °C with 5% CO_2_ in a humidified atmosphere. SH-SY5Y, SK-N-SH, SK-N-SHm, their respective FACS-sorted populations and their xenografts were cultured in DMEM high glucose (Cat#D5796, Sigma) with 10% FBS (Cat#F7524, Sigma). Cells from neuroblastoma PDX models including IC-pPDXC-63 and IC-pPDXC-109 and their xenografts were cultured in RPMI-1640 (Cat#R8758, Sigma) with 10% FBS.

For reconstitution experiments after FACS sorts, 100,000 cells were plated in 24-well plates in triplicates, in their respective media. For PDX and xenografts culture, cells were obtained after “Tumor dissociation into single-cell suspension” (protocol described below) and one million cells were plated in six-well plates, in their respective media. When adherent cells reached confluence, they were trypsinized and analyzed by FACS at least once a week. All reconstitution experiments are made using 10% FBS, except in EGF/TNFα experiment in which we used 2% FBS (condition that did not alter cell viability) complemented with growth factors at 20 ng/mL final.

All cells were monthly checked by qPCR (Venor® GeM qEP 11-9250, Minerva biolabs®) for the absence of mycoplasma.

### Mouse xenograft experiments

Xenografts experiments and PDXs were performed in female Swiss Nude adult mice (Charles River Laboratories, strain code 620). After transfer, mice were housed for 2–3 weeks for acclimation, in the animal facility, in a room with a 12 h:12 h light/dark cycle, a temperature of 22 +/− 2 °C and a targeted hygrometry from 40 to 70%. At the time of injection or engraftment, the mice were at least 8 weeks old.

For each cell line, 0.5 million cells were injected subcutaneously in the flanks of Nude mice with a ratio of 50/50 standard medium (DMEM: Cat# SH30022.01, GE Healthcare) and BD Matrigel™ (Cat# 356234, BD Biosciences). Tumor volume was measured every 2 or 3 days with a caliper, calculated as *V* = (*a*/2) * *b* * ((*a* + *b*)/2), a and b being the largest and smallest diameters, respectively. Mice were sacrificed when the tumor reached 2500 mm^3,^ the maximal tumor size permitted by the ethics committee. In vivo experiments for this study were performed in accordance with the recommendations of the European Community for the care and use of laboratory animals (2010/63/UE). Experimental procedures were specifically approved by the ethics committee of the Institut Curie CEEA-IC #118 (Authorization APAFIS#11206-2017090816044613-v2 and APAFIS#34207-2021120215196250-v1 given by National Authority) in compliance with the international guidelines.

### Patient-derived Xenografts (PDX models)

Written informed consents for the establishment of PDXs were obtained for all patients from parents or guardians. GR-NB4 (previously named MAP-GR-A99-NB-1^17^), GR-NB5 (previously named MAP-GR-B25-NB-1^17^), GR-NB7 and GR-NB10 have been provided by Birgit Geoerger (Gustave Roussy, Villejuif, France). IC-pPDX-63, IC-pPDX-75, IC-pPDX-109, IC-pPDX-112, IC-pPDX-196, and IC-pPDX-197 have been developed at Institut Curie. HSJD-NB-003, HSJD-NB-004, HSJD-NB-005, HSJD-NB-009, and HSJD-NB-011 PDX models have been provided by Angel Carcaboso (Institut de Recerca San Joan de Déu, Barcelona, Spain). These models have been generated from patients under an Institutional Review Board-approved protocol (OBS170323CPP ref3272; dossier No. 2015-A00464-45) or within a clinical trial (Supplementary Table 2).

### Neuroblastoma patients

Neuroblastoma samples for single-cell analyses were obtained from patients treated at Institut Curie. Surplus tissues obtained at diagnosis or relapse were processed immediately after receipt at the laboratory for molecular diagnosis (Unité de Génétique Somatique). Written informed consents for this study, including the analysis of surplus tumor tissue were obtained for all patients from parents or guardians. Out of the 18 studied patients (Supplementary Table 1), six patients were enrolled in the MICCHADO study (ClinicalTrials.gov identifier NCT03496402) and four patients in the MAPPYACTS trial (ClinicalTrials.gov identifier NCT02613962), with two patients enrolled in both programs. Within these studies, approval of this research was given by the decision of the ethics committees Sud Est VI, reference AU 1388, and Ile de France III, reference Am7158-2-3272. The study was approved by the Institut Curie’s Institutional Review Board (CRI-DATA220185).

### Methods details

#### Cell identity

Noradrenergic and mesenchymal tumor cell identities were defined using the transcription factors (TFs) previously identified^17,18^. This common list of 53 TFs is composed of 19 noradrenergic (NOR TFs) and 34 mesenchymal (MES TFs). They have been used for hierarchical clustering of bulk RNAseq data and as signatures in single-cell RNAseq data.

The tumor cell identity score was calculated as the difference between mesenchymal and noradrenergic scores, each score being the mean expression of the transcription factors associated to each identity.

### Immunofluorescence

100,000 cells for SK-N-SH, SK-N-SHm and IC-pPDXC-63 cell lines were plated in a four-well Lab-Tek chamber (Cat# 177399PK, Thermo Fisher) 48 h before immunostaining. Cells were fixed with 4% PFA buffer, permeabilized with a 0.2% triton solution and blocked in a 1% BSA 0.1% triton solution and incubated with anti-PHOX2B (Santa Cruz Biotechnology Cat# sc-376997, RRID:AB_2813765) and anti-CD44 (Cat# 15675-1-AP, Proteintech) at 1:100. Secondary antibodies were Cy5-Anti-Mouse (Cat# 715-175-151, Jackson ImmunoResearch Labs/1:100, RRID:AB_2340820) and Cy3-Anti-Rabbit (Cat# 711-165-152, Jackson ImmunoResearch Labs/1:100, RRID:AB_2307443) and DAPI (Cat# 62248, Thermo Fisher) was diluted at 1:1000 in ProLong™ Gold (Cat# P36930, Thermo Fisher).

### Chemotherapy treatments

IC-pPDXC-63 and SK-N-SH cell lines were plated in 96-well plates 24 h before the addition of doxorubicin or etoposide. Seeding densities for each cell line were optimized to reach 80% confluence in the untreated cells. Cells were treated with chemotherapeutic agents for 72 h. Cell viability was then measured using the Resazurin reagent (R7017, Sigma-Aldrich).

### RNA-sequencing and analyses

RNAs were extracted from frozen tumors by mechanical crushing followed by TRIzol® reagent (Cat#15596018, Invitrogen) and purified with the NucleoSpin RNA kit (Cat# 740955.50, Macherey-Nagel). For the bulk single-cell RNA-seq samples and the cell lines, extraction and purification were done directly using this NucleoSpin RNA kit according to manufacturer recommendation. RNA quality was assessed with a Bioanalyzer instrument and RNAs with an RNA Integrity Number above 7 were processed for sequencing. RNA sequencing libraries were prepared from 500 ng to 1 μg of total RNA using the Illumina TruSeq Stranded mRNA Library preparation kit according to manufacturer recommendation. For the xenografts of IC-pPDXC-63 in vivo sample, mRNA Library preparation was done with TruSeq RNA Exome from Illumina. 100 bp paired-end sequencing was performed with the Illumina NovaSeq 6000 instrument.

Reads were aligned to the human reference genome hg38/GRCh38 using STAR 2.6.1a_08-27 (RRID:SCR_015899, https://github.com/alexdobin/STAR) with the following options: outFilterMismatchNoverLmax 0.04, alignIntronMin 20, lignIntronMax 1000000, outFilterMultimapNmax 20. Gene expression values (FPKM = fragments per kilobase per million reads) were computed by Cufflinks v2.2.1 (RRID:SCR_014597, http://cole-trapnell-lab.github.io/cufflinks/) and further normalization between samples was done using quantile normalization (R/LIMMA v3.30.7 (RRID:SCR_010943)).

For scatterplots confronting gene expression and tumor cell identity score, we used the bulk RNAseq of 25 neuroblastoma cell lines previously generated^17^.

### PHOX2B immunohistochemistry

Tumors were fixed in a 4% formol buffer (VWR) during 24 hours, embedded in paraffin and cut in 4 µm slices. For PHOX2B immunohistochemistry, the REAL™ EnVision™ Detection System (Cat# K406511-2, Agilent Technologies) was used and the antibody against PHOX2B (Cat# sc-376997, B-11, Santa Cruz) was diluted at 1:200. Xenograft slices were also colored with a hematoxylin solution.

### ChIP-seq and analyses

Cells from neuroblastoma cell lines and from frozen tissues were fixed with formaldehyde 1% during 10 and 8 min respectively, then subjected to lysis and chromatin shearing with Bioruptor®sonicator from Diagenode. H3K27ac chromatin immunoprecipitation (ChIP) experiments were performed using the iDeal ChIP-seq kit for histones (Cat# C01010171, Diagenode) according to manufacturer recommendations with 1 million cells per IP and the H3K27ac rabbit polyclonal antibody (Abcam Cat# ab4729, RRID:AB_2118291, 1 µg per IP). Illumina sequencing libraries were prepared from the ChIP and input DNA using the TruSeq ChIP library preparation kit (cat# IP-202-1012, Illumina) according to the manufacturer’s protocol and sequenced on the Illumina NovaSeq 6000 instrument (single reads, 100 nt). ChIP-seq reads were mapped to the human reference genome hg19/GRCh37 using Bowtie2 v2.2.9 (http://bowtie-bio.sourceforge.net/bowtie2/index.shtml). Low quality reads were filtered using Samtools v1.9 (Q < 20). Peaks were called with HMCan v1.40 (RRID:SCR_010858)^67^. Super-enhancers were called with LILY software^17^. LILY was also used to normalize HMCan density profiles between samples. The H3K27ac signal on super-enhancers shown in the heatmap was computed as the sum of normalized H3K27ac densities divided by the length of the super-enhancers. For transcription factors associated with several super-enhancers, the signal of the associated super-enhancers is summed and divided by their total length. PCA was performed on log_2_ values of super-enhancers scores of 5975 super-enhancers previously identified in the Supplementary Table 3 of Boeva et al.^17^.

### Flow cytometry analysis and sorting

Flow cytometry analysis was performed with the BD™ LSRII cytometer. Cells were detached with TrypLE™ Express Enzyme (Cat# 12604013, Gibco) and suspended in PBS. Cells were stained with Live/Dead fixable dead cell stain kit (Cat#L34955, Invitrogen) and permeabilized with the IntraPrep kit (Cat# A07803, BeckmanCoulter). The cell suspension was incubated with PHOX2B [Clone B-11] - AlexaFluor® 647 (Cat# SC-376997 AF647, Santa Cruz) and CD44-FITC (Cat# 338804, Biolegend) antibodies diluted at 1/100 during 30 min at 4 °C in dark. Gating strategy is illustrated in Fig. S2A.

Flow cytometry sorting was performed with the SH800 cell sorter (Sony). Cells were detached with TrypLE™ Express Enzyme (Cat# 12604013, Gibco), suspended in PBS and incubated with CD44-FITC antibody diluted at 1/100 during 30 min at 4 °C in dark. DAPI (Cat#62248, ThermoScientific) was used at 1/1000 to exclude dead/dying cells. The first gating for sort was based on FSC/SSC and represents 60% for IC-pPDXC-63 and 75% for SK-N-SH. Doublet cells were eliminated by gating on SSC-W/SSC-H followed by FSC-W/FSC- H. The second gating based on DAPI negative staining eliminated dead/dying cells. The boundaries between positive staining and negative staining were always more than 1 Log of fluorescence intensity (Fig. S2B). A control tube without staining was always analyzed to determine auto-fluorescence.

### Tumor dissociation into single-cell suspension

Patient samples (biopsies and surgical resections) and PDX tumors were cut with scalpels in small fragments. Enzymatic dissociation was realized in CO_2_ independent medium (GIBCO) containing 150 µg/mL Liberase™ TL Research Grade (Cat# 5401020001, Merk) and 150 µg/mL DNase (DN25, Sigma Aldrich), for 30 min at 37 °C with 400 rpm agitation. Cell suspension was then filtered using 70 µm cell strainer (Cat# 130-098-462, Miltenyi Biotec). The cell suspension was washed twice with PBS. Viability was measured using Vi-cell XR Viability Analyzer (Beckman Coulter). For some PDXs, the Mouse Cell Depletion Kit was used following the manufacturer’s instructions (Cat# 130-104-694, Miltenyi Biotec).

### Single-cell RNA-sequencing experiments and preprocessing of data

Single-cell RNA-seq was performed with the 10x Genomics Chromium Single Cell 3’ Kit (v3) according to the standard protocol. Libraries were sequenced on an Illumina HiSeq2500 or NovaSeq 6000 sequencing platform. CellRanger version 3.1.0 (10x Genomics, https://support.10xgenomics.com/) was used to demultiplex, align and generate UMI count tables from sequencing reads. Two reference genomes were used to align reads:

• The human reference genome (hg38/GRCh38) for the 18 patient samples and for in vitro cell lines.

• A human-mouse reference genome (GRCh38-mm10) for the 15 neuroblastoma PDX models. In this scenario, we identified the mouse and human cells after inspection of the percentage of coverage from GRCh38 genome. We labeled cells as either human (at least 80%), murine (<30%) or human-murine doublets (between 30 and 80%). Only human and human-murine doublet cells were selected and coverage plus gene information of only GRCh38 genome were retained for downstream analysis. Summary of analyses are shown in Supplementary Tables 3 and 4. Of note, we performed two biological replicates for three models: HSJD-NB-005, IC-pPDX-63 and IC-pPDX-75 (* in Supplementary Table 4) to assess reproducibility at distinct passages in mice.

### Ambient mRNA correction

SoupX R package v1.4.5 (https://github.com/constantAmateur/SoupX) was used to estimate and correct for ambient mRNA contaminations in samples aligned to GRCh38 genome. First, depending on the expression profile of empty droplets (defined as droplets with UMI count <100), we picked, as recommended, either *HBB/HBA* or immunoglobulin genes (*IGHA1, IGHA2, IGHG1, IGHG2, IGHG3, IGHG4, IGHD, IGHE, IGHM, IGLC1, IGLC2, GLC3, IGLC4, IGLC5, IGLC6, IGLC7, IGKC*) as marker genes to estimate contamination fractions. When these genes were absent from the ambient profile (the top 100 covered genes), we used the automatic mode provided by SoupX to estimate contamination fractions and generate corrected expression matrices. Summary of analyses is shown in Supplementary Table 3.

### Doublet detection

Scrublet^68^ v0.2.1 was used to detect potential doublets using default parameters (expected_doublet_rate=0.06). Cells marked as doublets were removed from subsequent analysis (results in Supplementary Table 3). Doublet detection was only feasible on samples aligned to GRCh38 genome.

### Quality control of single-cell data

First, all ribosomal genes (defined as *RLP/RPS* genes) were removed from the raw expression matrices. Then, coverage thresholds were set for each sample individually; an upper threshold was set to remove outlier cells with coverage greater than the 99th percentile, and a lower threshold was set to remove low quality cells with coverage inferior to the 1st percentile. To avoid cells with low number of genes, the same lower threshold was applied also on the number of genes thus defining a minimum number of genes required. An exception was made for the IC-pPDXC-63 and SK-N-SH cell lines for which the limits were 1000 UMI and 500 genes detected per cell. Finally, cells with more than 20% of reads mapping mitochondrial genes were removed.

### Normalization of single-cell data

Raw UMI counts were normalized using the SCTransform function of Seurat^69,70^ v3.1.5 (RRID:SCR_007322). Regressed variables included cell coverage, number of features, and the percentage of UMI from mitochondrial genes.

### Dimensionality reduction and cluster identification

Normalized count data was subjected to a PCA dimensionality reduction in which the first fifty principal components were computed. Uniform Manifold Approximation and Projections (umap) embeddings were calculated using the first thirty principal components as input and cells were clustered using the FindClusters function of Seurat.

### Cell type annotation

Marker genes that define cell clusters were identified after differential expression analysis using Seurat’s FindAllMarkers function. Clusters were annotated by comparing their top marker genes to canonical cell type markers from the literature.

### Generation of single-cell signature scores

To plot the expression of gene signatures in single cells, we used the AddModuleScore function from Seurat R package with 100 genes in the control gene set. Expression scale was binned into 10 bins when a gene signature is plotted and into 3 bins (1 = low, 2 = median, 3 = high) when a single gene is plotted.

### Single-cell RNA-seq data integrations

#### Patient tumor integration and dissection of tumor heterogeneity

Seurat v4.3.0 was used to integrate the 18 neuroblastoma patient samples using 2000 anchor features. The integrated object was subjected to dimension reduction and clustering as described above. Tumor cells and microenvironment were identified based on the expression of specific marker genes. To further dissect tumor heterogeneity, 34,292 tumor cells were extracted from the integration by InferCNV (see “Patient tumor cells extraction”), then Harmony^32^ (https://github.com/immunogenomics/harmony) was used for data integration.

#### PDX

Harmony v1.0 was used to integrated the 58,120 human cells coming from 15 PDX models. First, samples were preprocessed as described above, then Harmony was used for data integration. Of note, when several single-cell transcriptomes are available for the same model, only one was used for the integration to avoid over-representation.

For both patient tumors and PDX integrations, downstream analysis was carried out as described in Dimensionality reduction and cluster identification.

#### Independent published dataset from Jansky et al^13^

Starting from the published integrated and annotated dataset (64.769 neuroblastoma cells from 14 patients), raw counts were split per patient into individual objects using Seurat. Ribosomal genes were removed from raw counts and single objects were analysed individually as described in Normalization of single-cell data and Dimensionality reduction and cluster identification with cell coverage and number of features as regressed variables. Then, Seurat was used to re-integrate the normalized samples as in Patient tumor integration. Following the integration, clusters from the microenvironment were excluded along with cells previously annotated as Liver cells and the remaining cells (*n* = 59.335) were re-integrated and re-clustered using Seurat as described in this section.

### Patient tumor cell extraction

After integration of the 18 neuroblastoma single-cell RNAseq (from biopsies), immune cells from the microenvironment were excluded and the remaining cells (mixture of tumor cells, Schwann and mesenchymal cells) were filtered for cells expressing *HBB*, *PTPRC* or *IGCK* genes (expression > Q1; *n* = 1309). Then, cells were grouped by patient and copy number variations were called at the single cell level for each group using InferCNV as previously described. In this case, InferCNV failed to segregate cells into normal and tumor cells for all groups using a hierarchical clustering (option group_by_cluster=FALSE). Therefore, single cell malignancy was assessed based on the distribution of the average residual expression values over a given chromosome showing an emblematic copy number variation (17q gain or 1p loss). After careful examination, a threshold was set on each distribution to distinguish values that likely correspond to the observed gain or loss. These thresholds were used to select 34,647 cells. A final step of filtering based on InferCNV hierarchical clustering was necessary to remove persistent normal cells (*n* = 355) resulting in the final set of patient tumor cells (*n* = 34,292 cells) used to dissect tumor heterogeneity.

### Cell cycle analysis

We scored single cells based on expression of G2/M and S phase markers using Seurat’s CellCycleScoring function.

### Copy number analysis in single cells

Copy number variations at the single cell level were called with R package InferCNV^71^ v1.9.1 (https://github.com/broadinstitute/inferCNV) using default parameters. Normal cells from the microenvironment of one case, TD3, (*n* = 282 cells) were used as reference cells for patient tumor samples, and monocytes from publicly available single cell RNA sequencing of healthy human PBMCs^72,73^ (GEO: GSE115189) were used as reference cells (*n* = 376 cells). Cells with fewer than 1000 UMI were excluded.

### Statistics and reproducibility

All experiments were successfully replicated. No data were excluded from the analyses. No statistical method was used to predetermine sample size. The experiments were not randomized. The Investigators were not blinded to allocation during experiments and outcome assessment. Statistical tests were performed using GraphPad Prism 8 (RRID:SCR_002798). Significance values and confidence intervals are described in the figure legends.

### Reporting summary

Further information on research design is available in the Nature Portfolio Reporting Summary linked to this article.

## Supplementary information

Supplementary information

Description of additional supplementary files

Supplementary Data 1 & 2

Reporting Summary

## Data Availability

Source data are provided with this paper as a Source Data file. Alignment of sequencing data were realized using the hg19/GRCh37, the hg38/GRCh38 and the mm10/GRCh38 public databases of the GRC website (https://www.ncbi.nlm.nih.gov/grc). ChIP-seq (RRID:SCR_001237) data of cell lines are available in Gene Expression Omnibus (GEO, RRID:SCR_005012) under the accession code GSE154907. All single-cell RNA-seq from biopsies and PDXs, RNA-seq and ChIP-seq data on the IC-pPDXC-63 cell line are available in European Genome-Phenome Archive (EGA) under the accession numbers EGAS00001004781 and EGAS00001005322. The study EGAS00001004781 contains three datasets: EGAD00001006557 (4 Fastq files of the ChIPseq done with the IC-pPDXC-63 cell line), EGAD00001007870 (22 BAM files corresponding to the scRNAseq done with PDXs and cell lines) and EGAD00001006558 (72 BAM and Fastq files of corresponding bulk RNAseq). The study EGAS00001005322 contains one dataset EGAD00001006556 which includes the 19 BAM files of the scRNAseq performed on neuroblastoma patient biopsies. Patient tumor bulk RNAseq have already been published^74^ and are accessible in EGA under the accession number EGAS00001007161. Access to EGA archive datasets is obtained by formal application to the Data Access Committee (DAC). Each DAC requires users/applicants to sign a Data Access Agreement (DAA), which details the terms and conditions of use for each dataset. Source data are provided with this paper.

## Source data

Source data

## Materials availability

Availability of the IC-pPDXC-63 and IC-pPDXC-109 models generated in this study is subjected to a Material Transfer Agreement.
