# Supplementary material for: Reversible transitions between noradrenergic and mesenchymal tumor identities define cell plasticity in neuroblastoma
Source: Nat Commun. 2023 May 4;14:2575. doi: 10.1038/s41467-023-38239-5 (PMC10160107; doi:10.1038/s41467-023-38239-5)
Supplement: Supplementary file 1 — Supplementary information [file 41467_2023_38239_MOESM1_ESM.pdf]

SUPPLEMENTARY INFORMATION

Reversible transitions between noradrenergic and mesenchymal tumor identities define cell plasticity in neuroblastoma

Authors: Cécile Thirant[1,2], Agathe Peltier[1,2], Simon Durand[1,2#], Amira Kramdi[1,2#], Caroline Louis-Brennetot[1,2#], Cécile Pierre-Eugène[1,2#], Margot Gautier[1,2], Ana Costa[1,2], Amandine Grelier[1,2], Sakina Zaïdi[1,2], Nadège Gruel[1,3], Irène Jimenez[2,3,4], Eve Lapouble[5], Gaëlle Pierron[5], Déborah Sitbon[5], Hervé J. Brisse[6], Arnaud Gauthier[7], Paul Fréneaux[7], Sandrine Grossetête[1,2], Laura G. Baudrin[8], Virginie Raynal[1,8], Sylvain Baulande[8], Angela Bellini[2,3,4], Jaydutt Bhalshankar[2,3,4], Angel M. Carcaboso[9], Birgit Geoerger[10], Hermann Rohrer[11], Didier Surdez[1,2], Valentina Boeva[12,13,14], Gudrun Schleiermacher[2,3,4], Olivier Delattre[1,2], Isabelle Janoueix-Lerosey[1,2*]

- Supplementary Figures 1 to 10

- Supplementary Tables 1 to 4

Supplementary Fig. 1

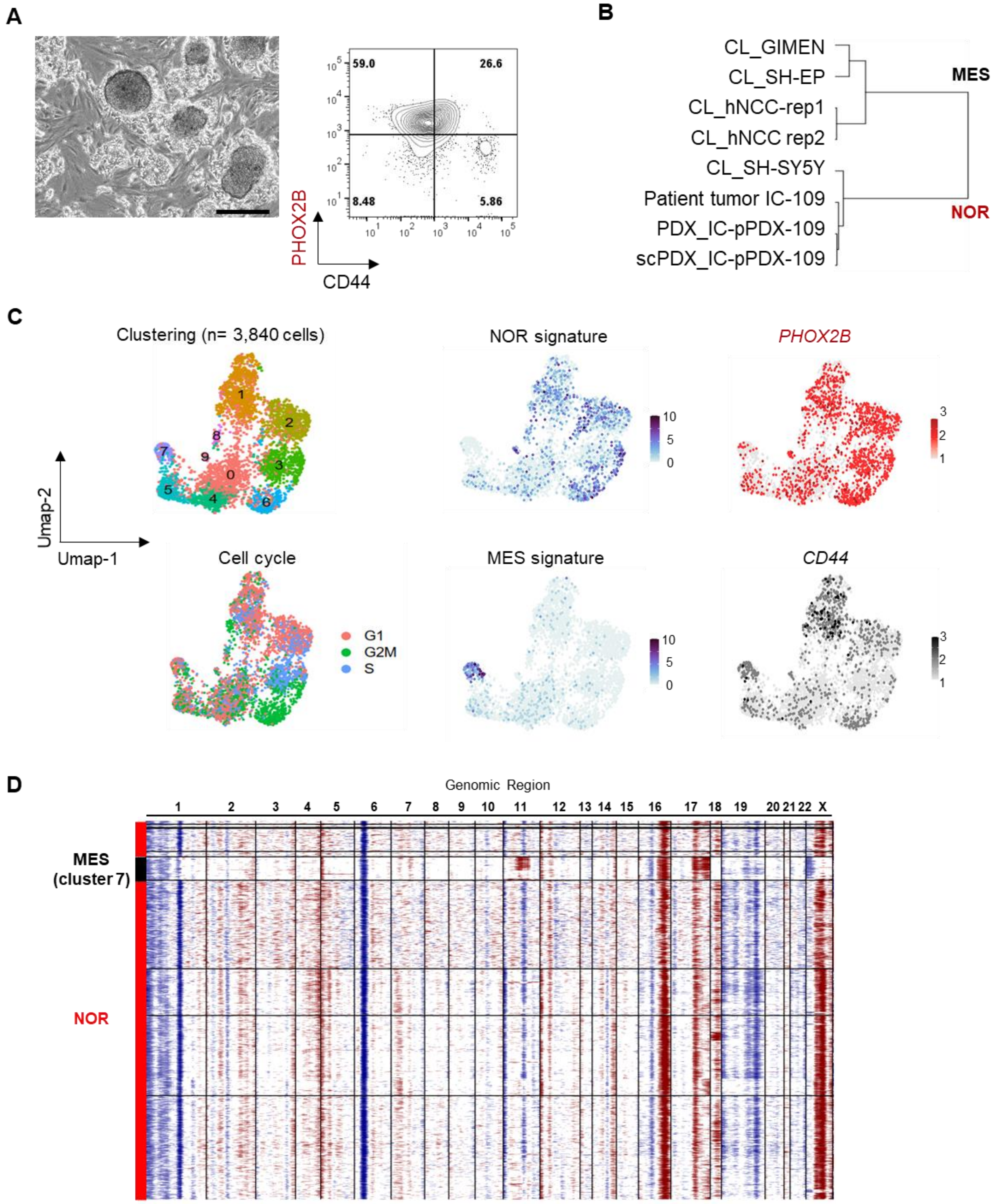

Supplementary Fig. 1: The IC-pPDX-109 model produces noradrenergic and mesenchymal tumor cells *in vitro* giving rise to the heterogeneous IC-PDXC-109 cell line. NOR = noradrenergic, MES = mesenchymal.

(**A**) Left: contrast phase microscopy photograph of the IC-pPDXC-109 cell line (scale bar = 50 µm, representative of 3 independent experiments). Right: FACS analysis using human PHOX2B and CD44 marker expression gated on live cells after doublet exclusion on the IC-pPDXC-109 cell line, representative of 3 independent experiments. (**B**) Unsupervised hierarchical clustering of bulk RNAseq data from cell lines (CL), PDX, single-cell data and original patient tumor, using the noradrenergic and mesenchymal transcription factor signatures[17,18] defining two branches. Source data are provided as a Source Data file. (**C**) Single-cell transcriptomic analyses of the IC-pPDXC-109 cell line by Seurat showing clustering at resolution 0.8 and cell cycle phases. Two main cell identities are highlighted by noradrenergic and mesenchymal transcription factor signatures[17,18] and *PHOX2B* and *CD44* expression, respectively. Each cell identity includes cycling cells. (**D**) Inferred genomic profile of the IC-pPDXC-109 cell line obtained with InferCNV on single-cell RNAseq data.

Supplementary Fig. 2

A

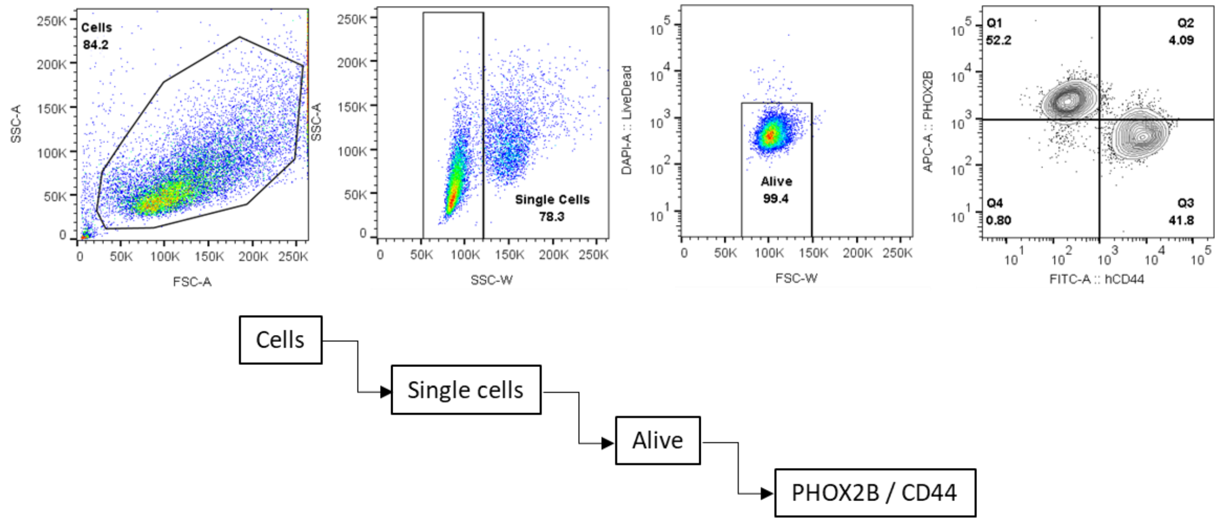

B

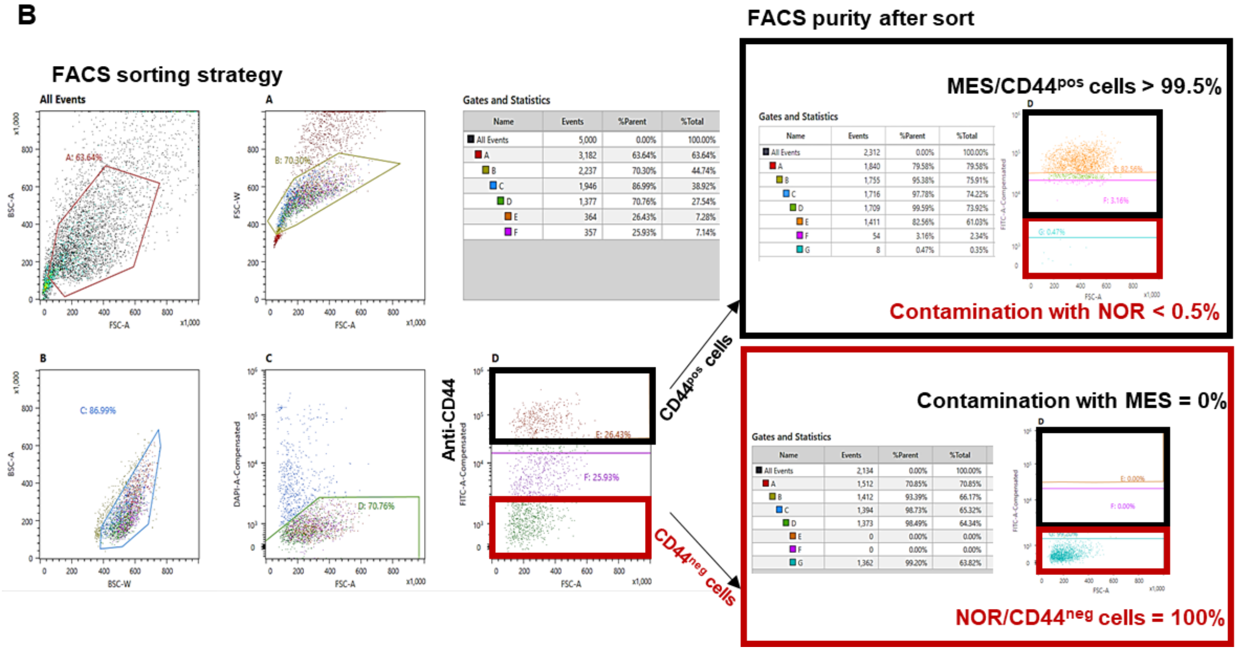

Supplementary Fig. 2

C

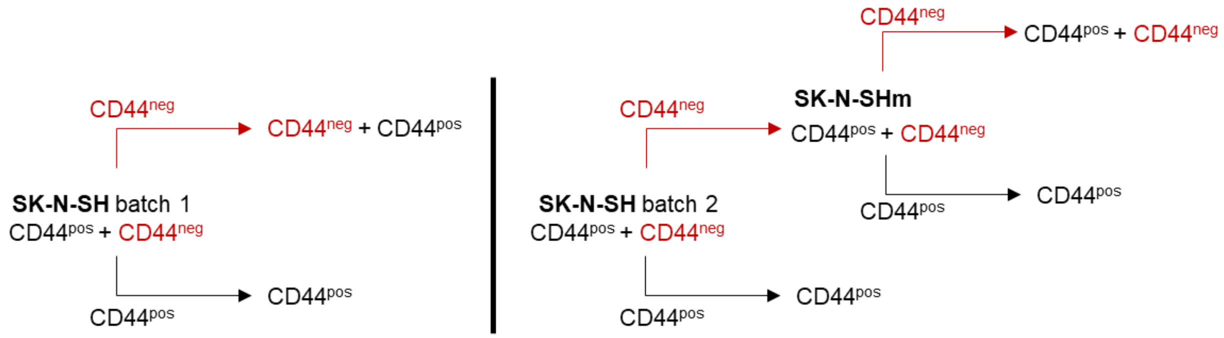

D

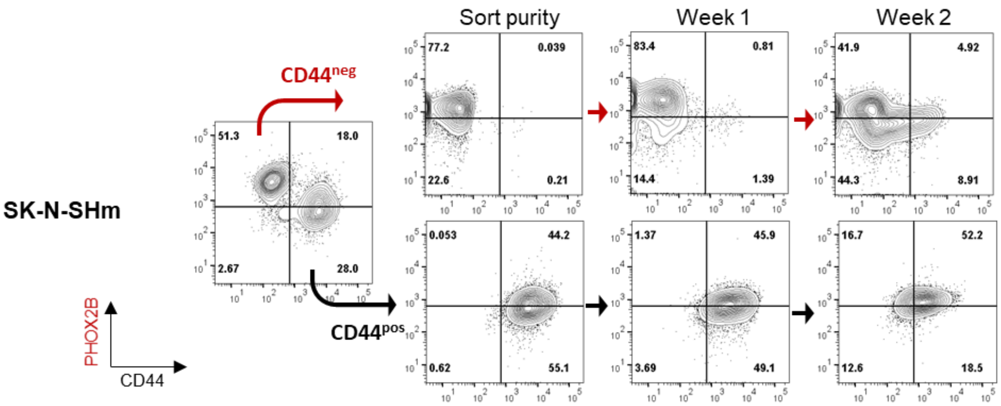

Supplementary Fig. 2: CD44 FACS sorting to study noradrenergic-mesenchymal plasticity in cellular models.

NOR = noradrenergic, MES = mesenchymal. (**A**) Example of the FACS gating strategy assessed to follow PHOX2B and CD44 expression gated on live cells after doublet exclusion. (**B**) Example of the FACS gating strategy and purity for CD44 cell sorts. Noradrenergic/CD44^{neg} cells were always 100% pure whereas mesenchymal /CD44^{pos} cells contain <0.5% of CD44^{neg} cells. (**C**) Schematic representation of reconstitution experiments done with the SK-N-SH model using several batches and CD44 cell sorts by FACS. (**D**) SK-N-SHm (from batch 2) CD44^{neg} and CD44^{pos} FACS-sorted cells are cultured and followed by FACS analysis using PHOX2B and CD44 marker expression gated on live cells after doublet exclusion for 2 weeks. Only CD44^{neg} cells are able to reconstitute a heterogeneous population of both noradrenergic/CD44^{neg} and mesenchymal /CD44^{pos} cells.

Supplementary Fig. 3

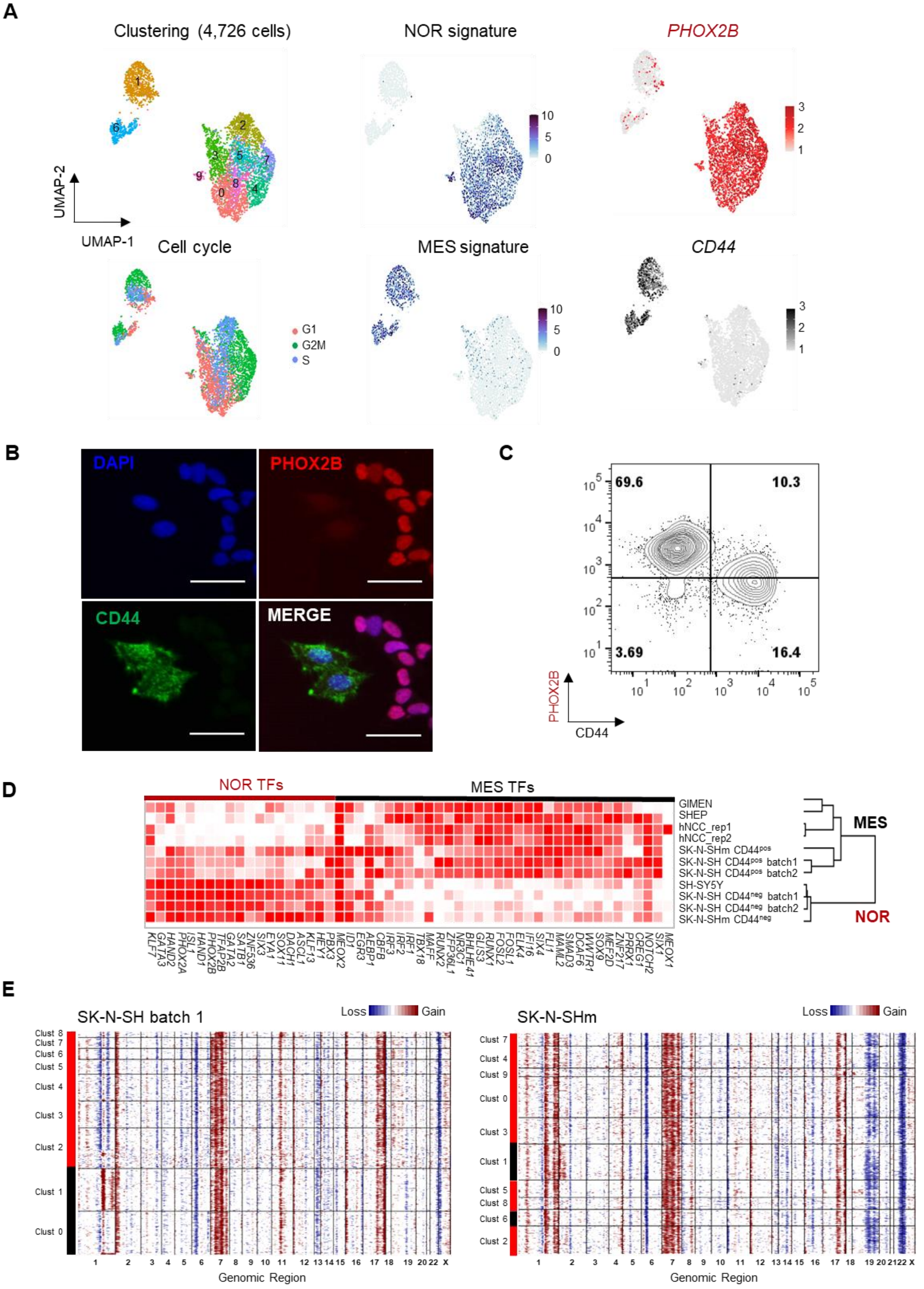

Supplementary Fig. 3: The SK-N-SHm cell line is a mixed cell line obtained from SK-N-SH CD44^{neg} cells. NOR = noradrenergic, MES = mesenchymal. (**A**) Single-cell RNA-seq analysis by Seurat of the SK-N-SHm cell line. The umap plot shows the clustering at resolution 0.8 and the cell cycle phases. Two main cell identities are highlighted by noradrenergic and mesenchymal transcription factor signatures[17,18], *PHOX2B* and *CD44* expression, respectively. Each cell identity includes cycling cells. (**B**) Immunofluorescence analyses of the SK-N-SHm cell line with the PHOX2B and CD44 markers (scale bar = 50 µm, representative of 3 independent experiments). (**C**) FACS analysis using human PHOX2B and CD44 marker expression gated on live cells after doublet exclusion on the SK-N-SHm cell line, representative of 3 independent experiments. (**D**) Heatmap showing the expression of noradrenergic and mesenchymal transcription factors (TFs)[17,18] in CD44^{pos} and CD44^{neg} cells of two batches of the SK-N-SH cell lines. Source data are provided as a Source Data file. (**E**) Genetic alterations were predicted from single-cell RNAseq data using the InferCNV tool, illustrating some genetic variability between different batches of SK-N-SH cell line.

Supplementary Fig. 4

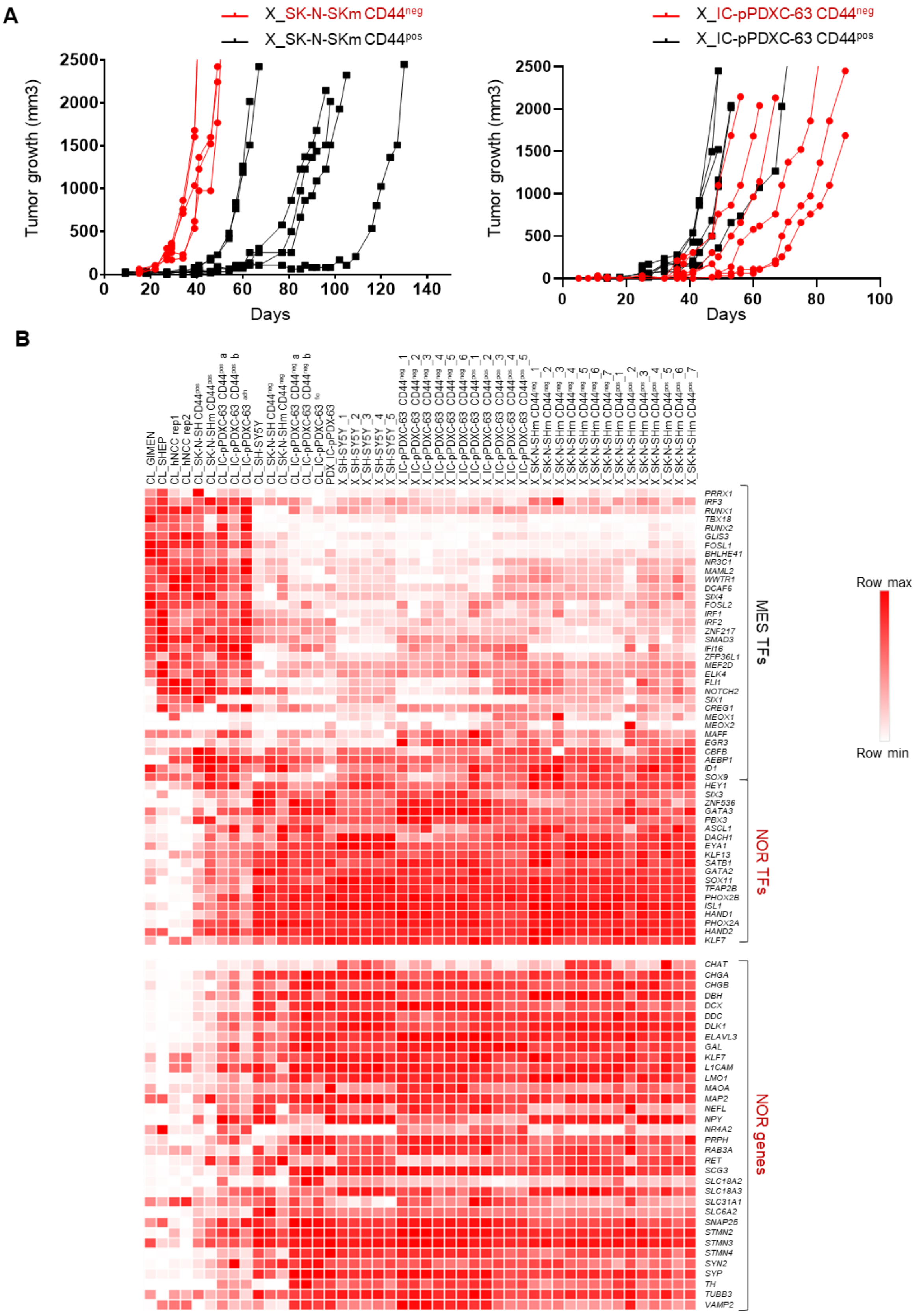

Supplementary Fig. 4: Mesenchymal tumor cells form noradrenergic tumors *in vivo*.

NOR = noradrenergic, MES = mesenchymal. Source data are provided as a Source Data file. (**A**) Individual tumor growth curves of SK-N-SHm $CD44^{pos}$ or $CD44^{neg}$ xenografts (n=7) and IC-pPDXC-63 $CD44^{pos}$ or $CD44^{neg}$ xenografts (n=6). Mice were sacrificed when tumors reached ethical size. P-values obtained with the Log-rank (Mantel-Cox) test. (**B**) Heatmap showing the expression of noradrenergic and mesenchymal transcription factors (TFs)[17,18], and noradrenergic genes on the bulk RNAseq data comparing transcriptome data of cell lines (CL_), PDX and xenografts (X_) including those obtained from $CD44^{pos}$ and $CD44^{neg}$ sorted cells.

Supplementary Fig. 5

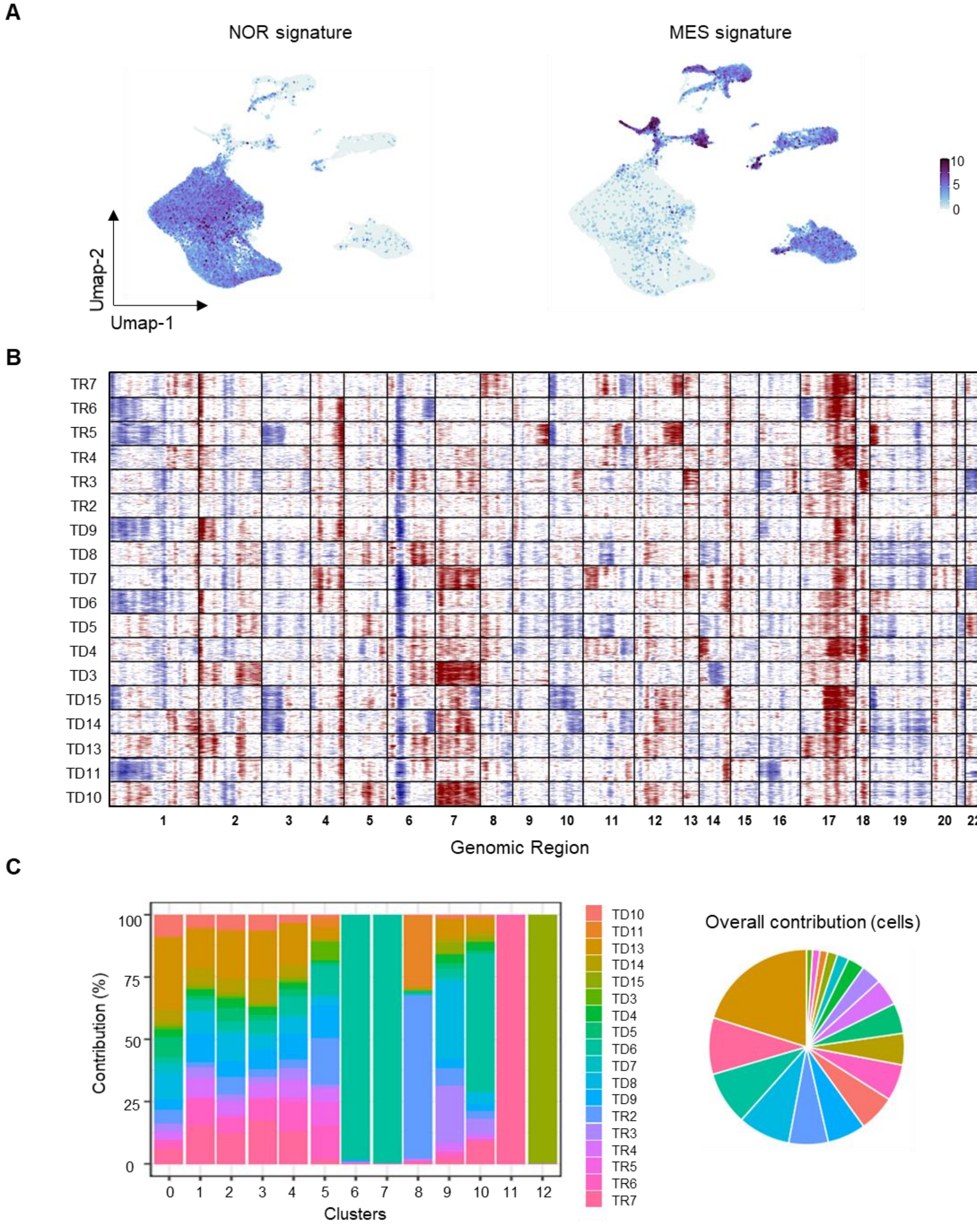

Supplementary Fig. 5

D

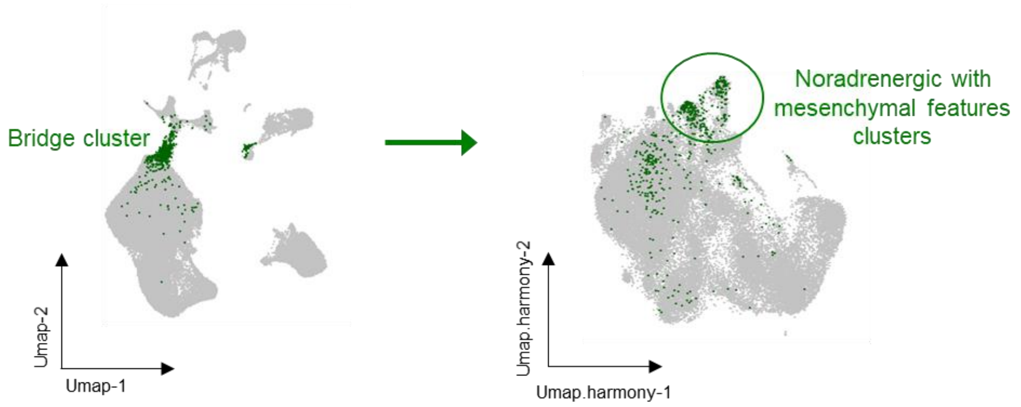

E

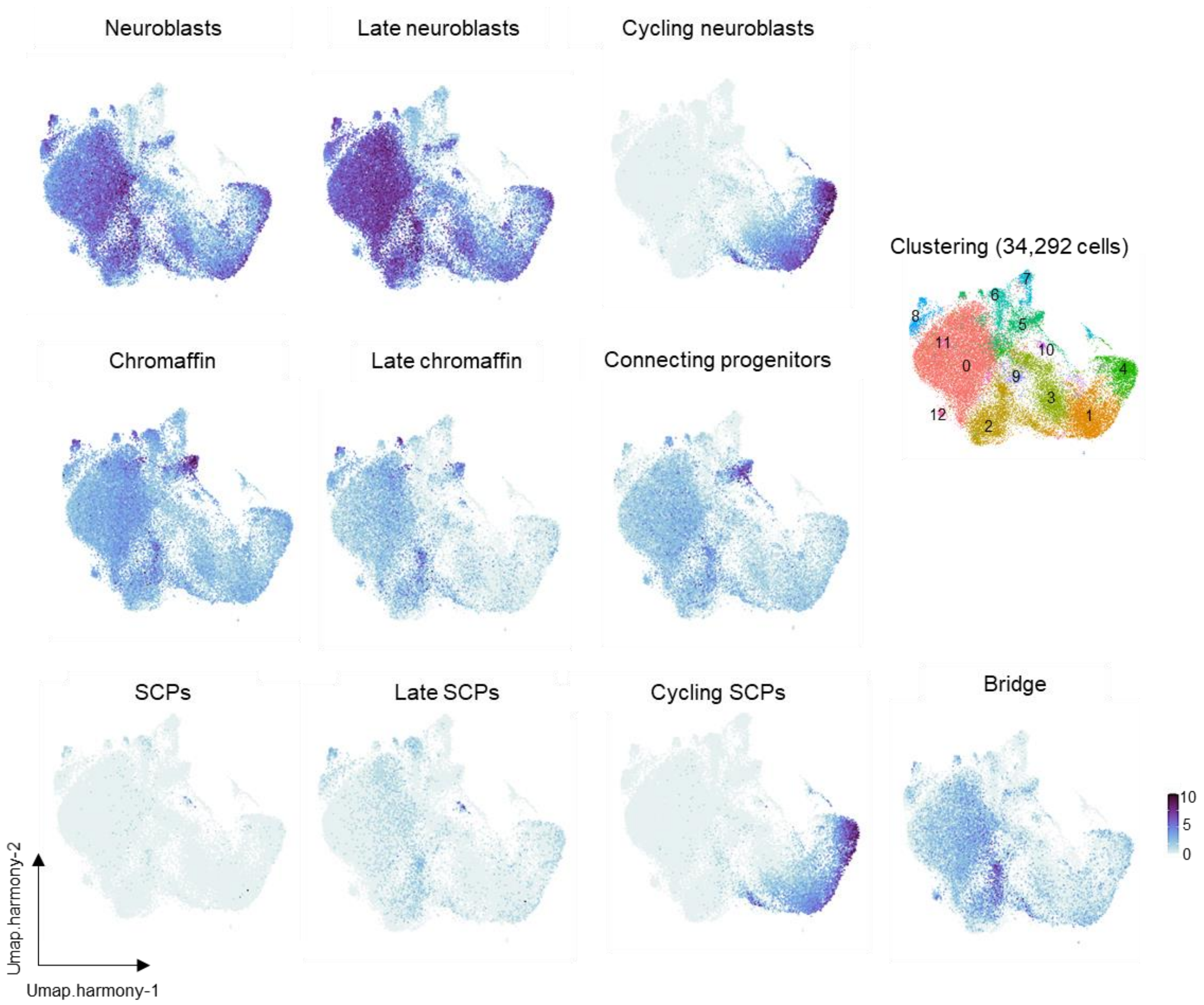

Supplementary Fig. 5: Detailed analyses of single-cell RNAseq of the 18 biopsies of neuroblastoma. NOR = noradrenergic, MES = mesenchymal. (**A**) Umap visualization of the noradrenergic and mesenchymal transcription factor signatures[17,18] plotted on the Seurat integration of 18 biopsies (n = 54,403 cells). (**B**) InferCNV analysis of 300 randomly-selected cells with high coverage (>1,000 UMI) from each sample. (**C**) Contribution of each case to clusters of the harmony integration of noradrenergic tumor cells only (n = 34,292 cells). The histogram highlights the contribution of the different samples to each cluster in percentage. The pie chart depicts the contribution of each sample to the total number of cells. Source data are provided as a Source Data file. (**D**) Highlight of the bridge cluster from the Seurat integration (n = 663 cells, left panel) into the Harmony integration of selected tumor cells (n= 561 cells, right panel). (**E**) Plots of normal adrenal developmental signatures[13] in the integration of noradrenergic tumor cells from the 18 biopsies.

Supplementary Fig. 6

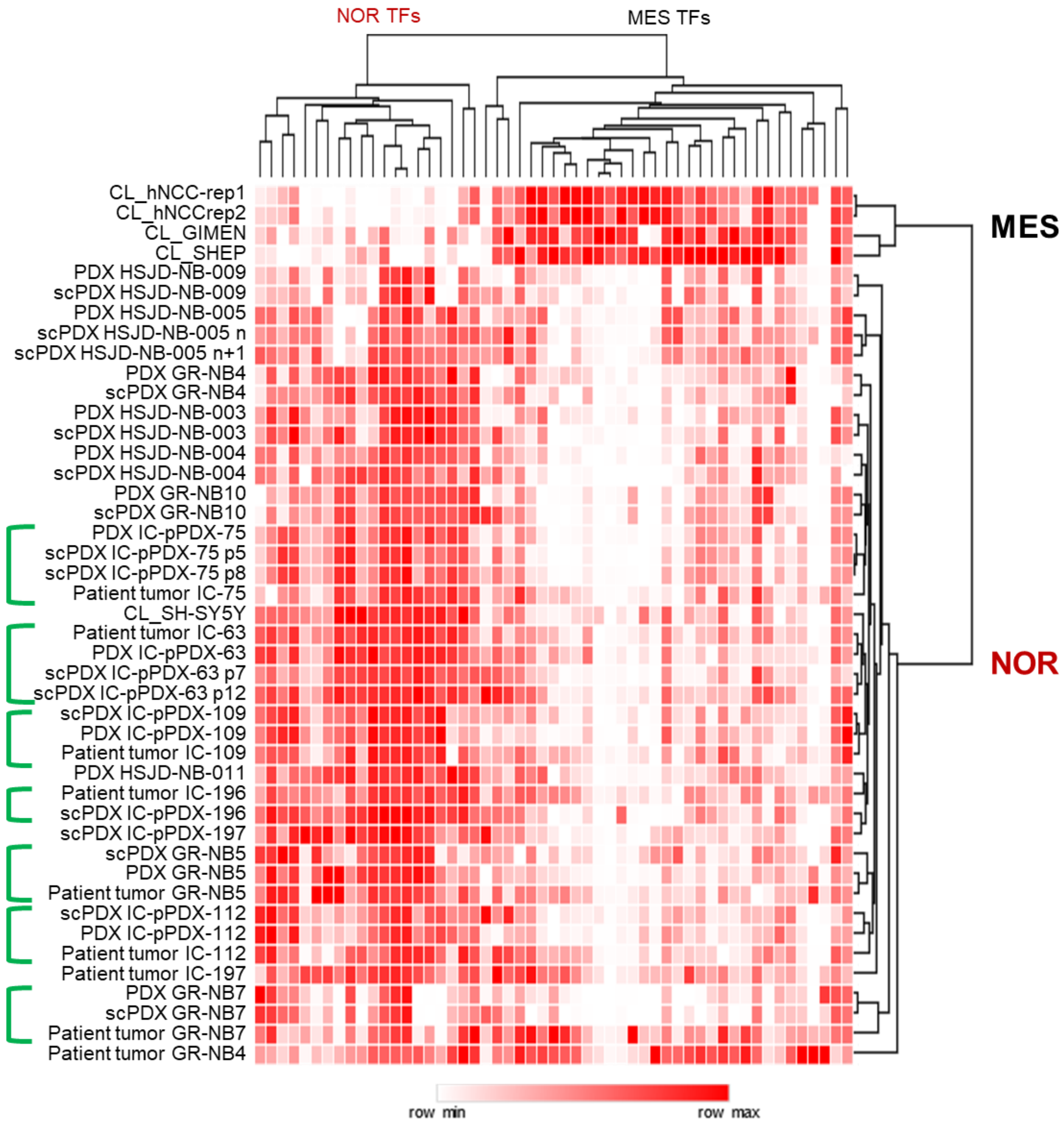

Supplementary Fig. 6: Relevance of PDXs as models of neuroblastoma pathology

NOR = noradrenergic, MES = mesenchymal. Unsupervised hierarchical clustering of genes and samples using bulk RNAseq obtained when available from the initial patient tumor biopsy (Patient tumor), its derived PDX model (PDX), and the preparation of cells used for single-cell RNAseq (scPDX) and some cell lines as references (CL), using expression of transcription factors (TFs) of the noradrenergic and mesenchymal identities [17,18]. Green brackets highlight a perfect clustering of the various samples of a specific patient. Source data are provided as a Source Data file.

Supplementary Fig. 7

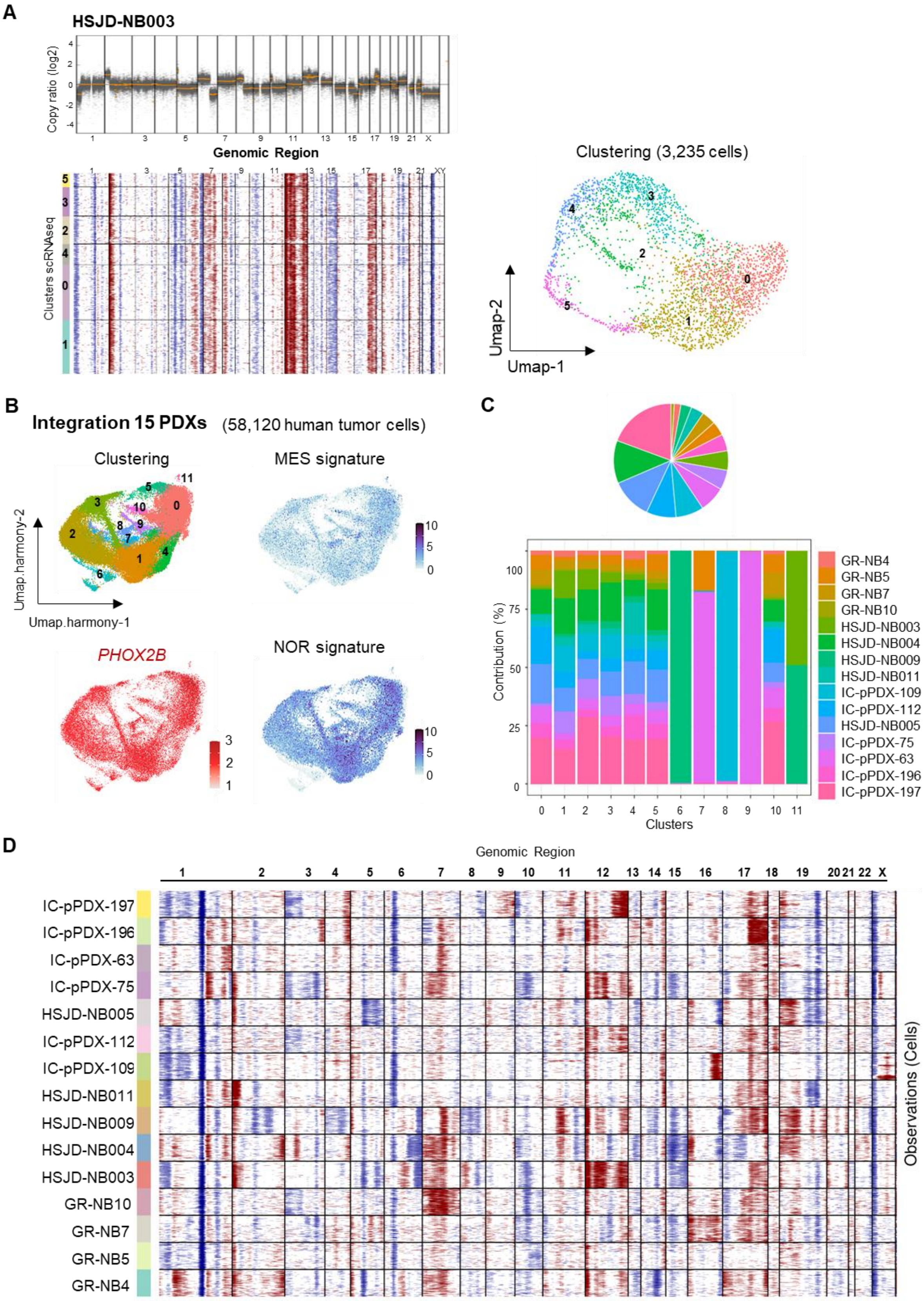

Supplementary Fig. 7: Detailed analyses of single-cell RNAseq of 15 neuroblastoma PDX models.

NOR = noradrenergic, MES = mesenchymal. (**A**) Top: Copy number profile inferred from WES data of HSJD-NB-003 PDX model. Bottom: InferCNV and Umap profiles obtained from scRNAseq data of HSJD-NB-003. (**B**) Umap of the 58,120 human tumor cells from the 15 PDX models obtained after the integration of scRNAseq by Harmony (clustering at resolution 0.3). The noradrenergic and the mesenchymal transcription factor signatures[17,18] have been plotted on the integration, as well as *PHOX2B* expression. (**C**) The pie chart depicts the contribution of each sample in the integration (total number of cells) and the histogram highlights the contribution of the different samples to each cluster in percentage. Source data are provided as a Source Data file. (**D**) Overview of the genomic alterations present in each case with the InferCNV profiles on the 15 PDX models, using 500 randomly-selected cells with high coverage (>1,000 UMI) from each sample.

Supplementary Fig. 8

A

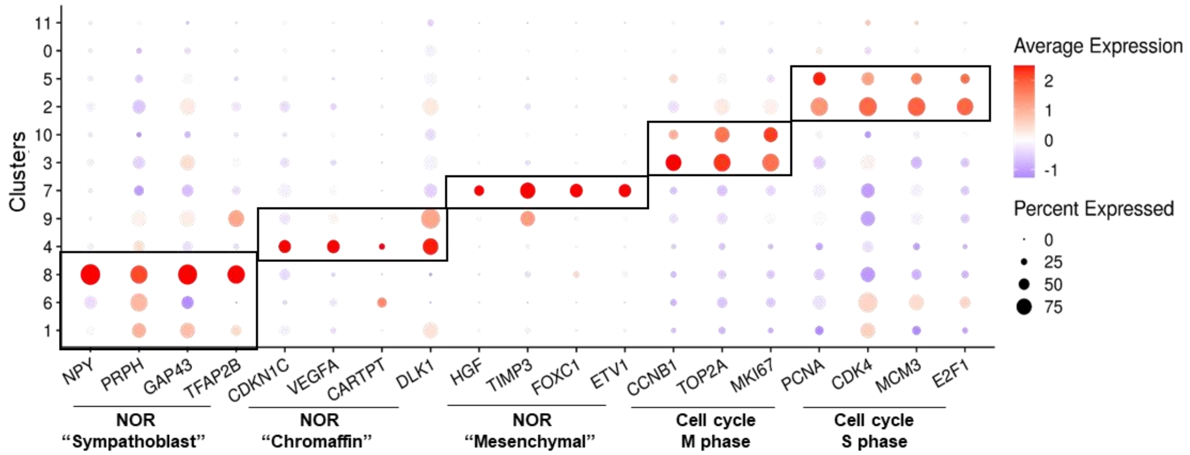

B

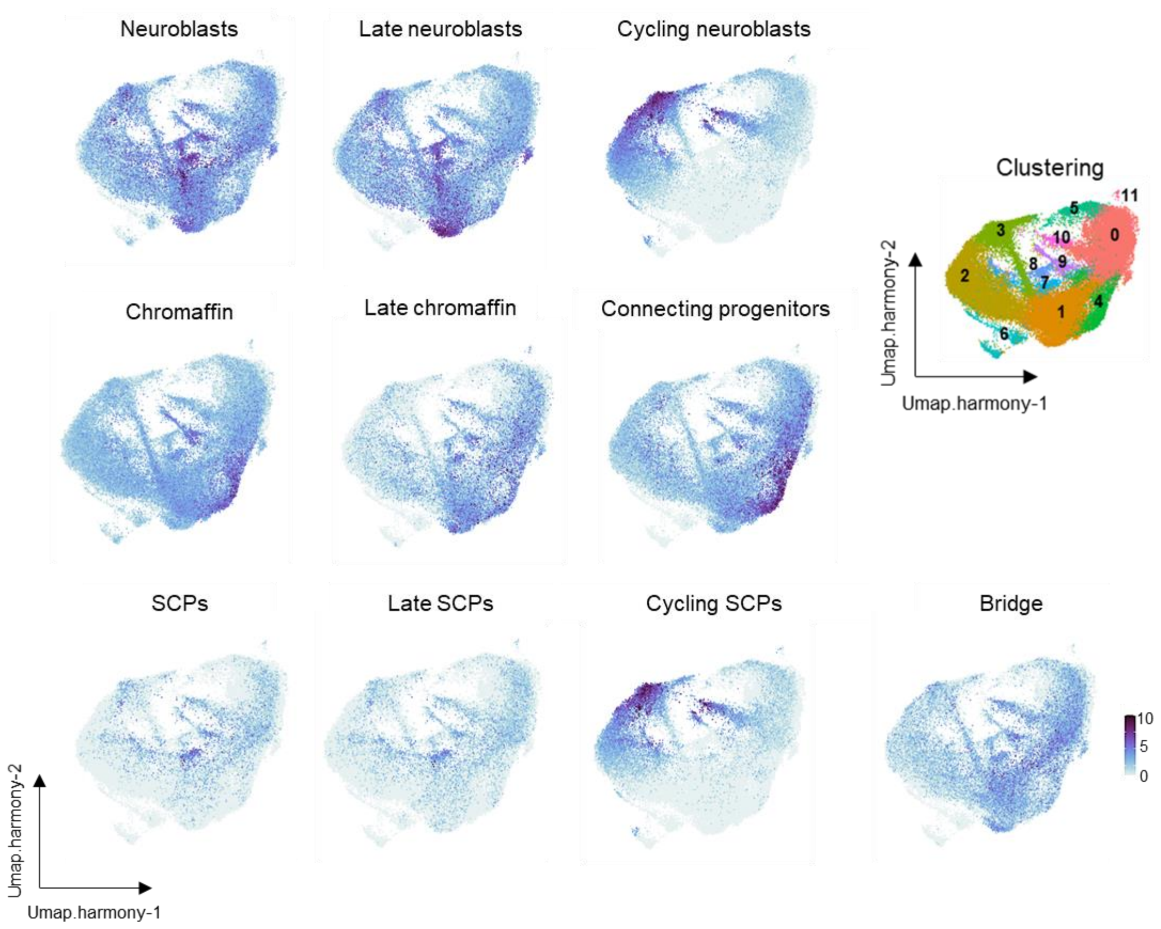

Supplementary Fig. 8

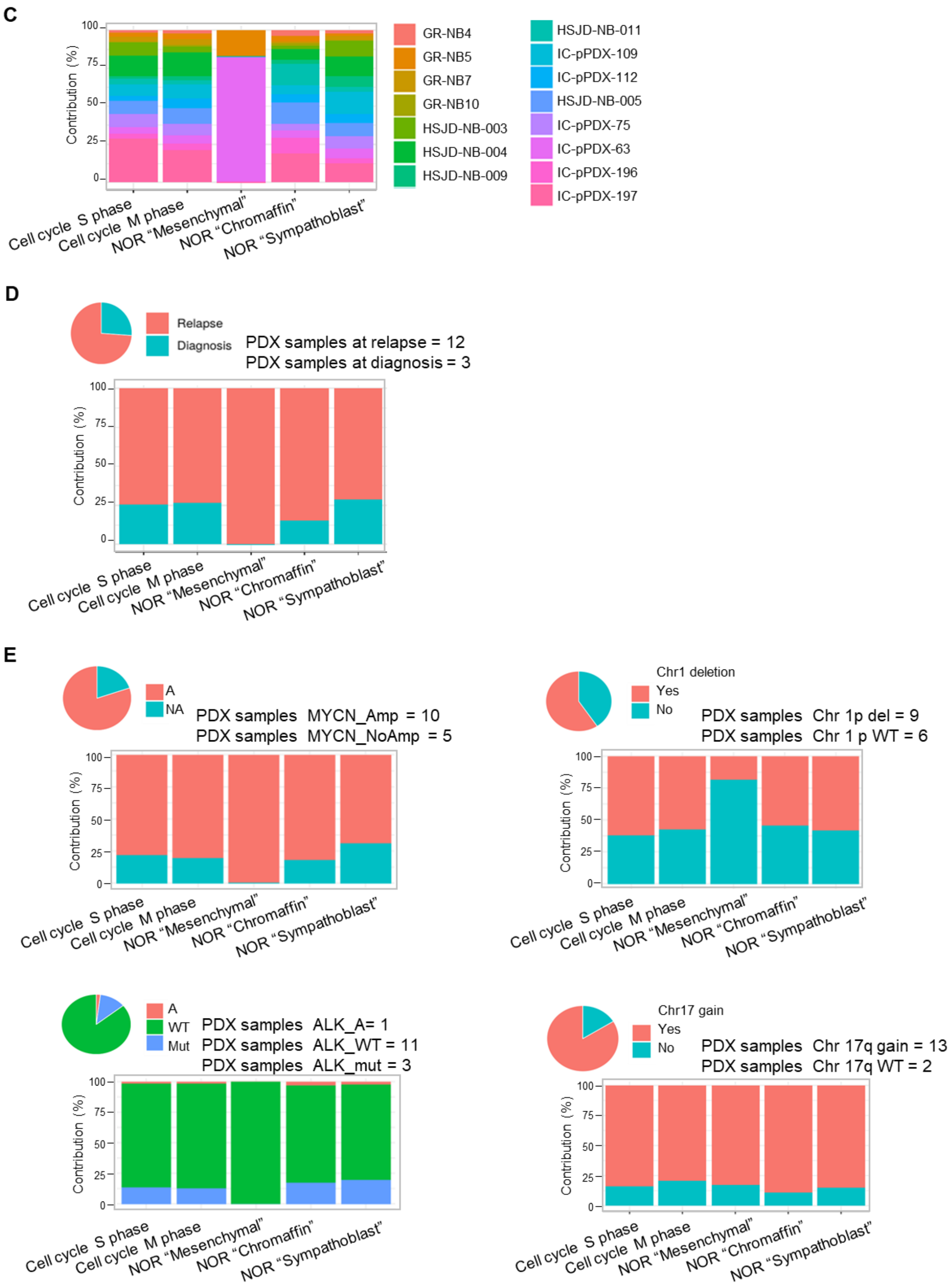

Supplementary Fig. 8: Intra-tumor heterogeneity assessed by scRNAseq in the neuroblastoma PDX cohort.

NOR = noradrenergic, SCPs = Schwann cell progenitors. Source data are provided as a Source Data file. (**A**) Dot plot graph illustrating cluster-specific gene expression in the integration of single-cell RNAseq from 15 neuroblastoma PDX models. Three main noradrenergic tumor cell identities could be defined. (**B**) Umap plots of normal adrenal developmental signatures[13] in the integration of 15 neuroblastoma PDX models. (**C**) Sample contribution to each cell identity. (**D**) Contribution of diagnosis versus relapse cases in the overall integration (pie chart) of the 15 neuroblastoma PDX models and in each cell identity. (**E**) Contribution of some classical neuroblastoma genomic alterations in the overall integration (pie chart) of the 15 neuroblastoma PDX models and in each cell identity.

Supplementary Fig. 9

A

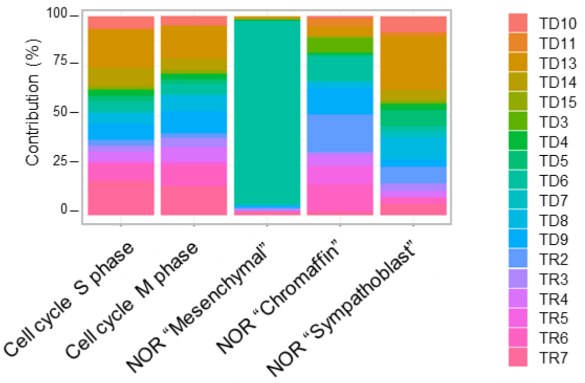

B

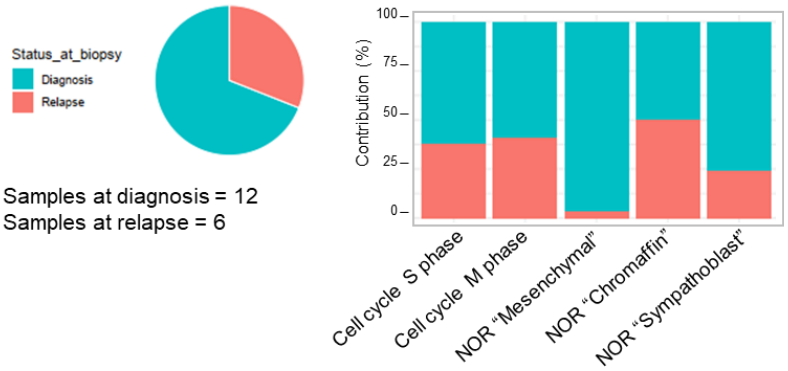

C

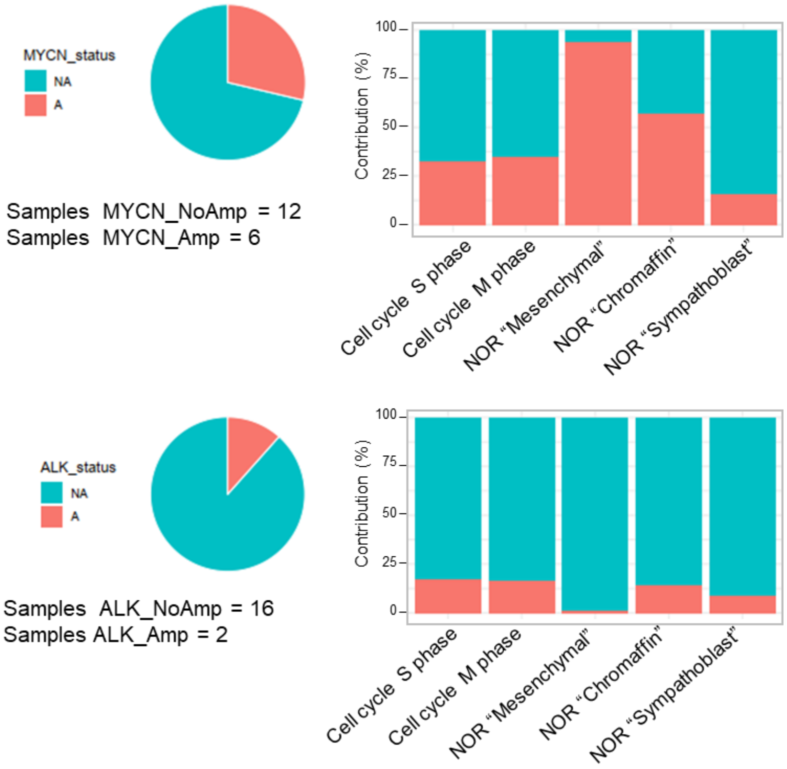

Supplementary Fig. 9: Intra-tumor heterogeneity assessed by scRNAseq in the neuroblastoma patient biopsies cohort.

NOR = noradrenergic. Source data are provided as a Source Data file. (**A**) Sample contribution to each cell identity. (**B**) Contribution of diagnosis versus relapse cases in the overall integration (pie chart) of the 18 neuroblastoma biopsies and in each cell identity. (**C**) Contribution of some classical neuroblastoma genomic alterations in the overall integration (pie chart) of the 18 neuroblastoma biopsies and in each cell identity.

Supplementary Fig. 10

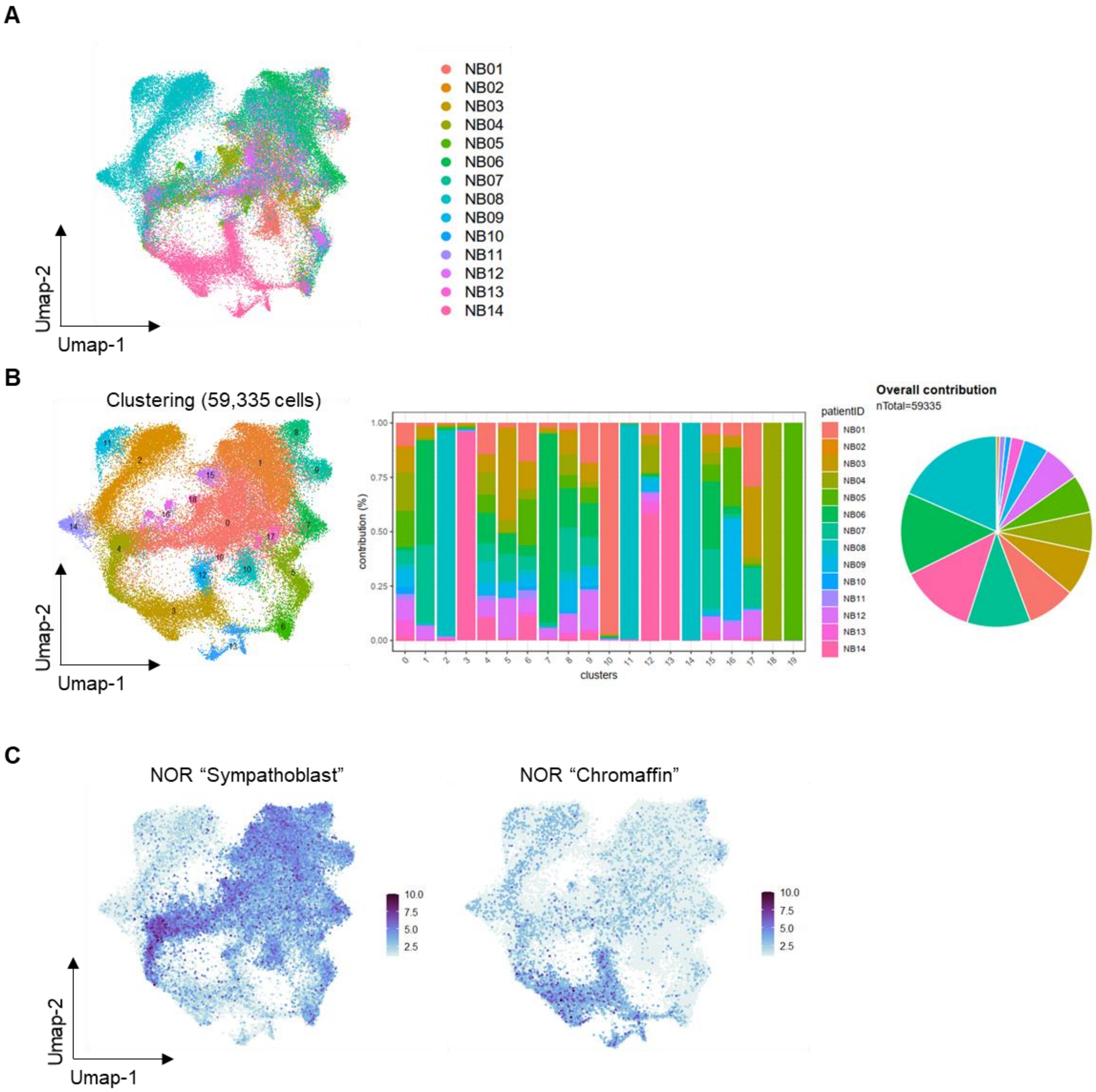

Supplementary Fig. 10: Intra-tumor heterogeneity in the snRNAseq dataset from Janksy *et al*.[13]

NOR = noradrenergic. (**A**) Seurat integration and contribution of noradrenergic tumor cells from 14 neuroblastoma cases (n = 59,335 cells). (**B**) Clustering and contribution of each case to clusters. Source data are provided as a Source Data file. (**C**) Plots of signatures identifying noradrenergic tumor cells with either sympathoblast or chromaffin features.

Supplementary Table 1: Characteristics of the 18 neuroblastoma biopsies studied by scRNA-seq.

Name	Analyzed at diagnosis or relapse	Primary tumor at diagnosis	INSS Stage	Age at diagnosis	Sex	MYCN status (A or NA)	ALK status (A or NA)	Genomic profile (NCA or SCA)	Site of biopy for scRNA-seq	Histology
TD3	Diagnosis	adrenal (right)	4	> 5y	M	A	NA	NCA	adrenal (right)	Neuroblastic tumor. MKI not determined.
TD4	Diagnosis	adrenal (left)	4	> 5y	M	NA	NA	SCA	adrenal (left)	Stroma-poor, poorly differentiated
TD5	Diagnosis	mediastinum with retroperitoneal	4	[18m - 5y]	F	NA	NA	NCA with atypical SCA	mediastinum (left)	Stroma-poor, poorly differentiated
TD6	Diagnosis	retroperitoneal (left)	3	[18m - 5y]	F	A	NA	SCA	retroperitoneal (left)	Stroma-poor, poorly differentiated
TD7	Diagnosis	mediastinal	4	[18m - 5y]	M	NA	NA	SCA	mediastinal	Stroma-poor, poorly differentiated
TD8	Diagnosis	mediastinal	Localized	< 18m	F	NA	NA	NCA	Thoracic (left)	Stroma-poor, poorly differentiated
TD9	Diagnosis	adrenal (left)	4	[18m - 5y]	M	A	A	SCA	retroperitoneal	Stroma-poor, poorly differentiated
TD10	Diagnosis	retroperitoneal (right)	Localized	< 18m	M	NA	NA	NCA	para-vertebral (right)	Stroma-poor, poorly differentiated
TD11*	Diagnosis	adrenal (right)	Localized	[18m - 5y]	M	NA	NA	SCA	adrenal (right)	Stroma-poor, poorly differentiated
TD13	Diagnosis	retroperitoneal-adrenal-	4S	< 18m	M	NA	NA	SCA	retroperitoneal (left)	Stroma-poor, poorly differentiated
TD14	Diagnosis	abdominal (median)	4	[18m - 5y]	M	A	A	SCA	retroperitoneal (left)	Poorly differentiated neuroblastoma or
TD15	Diagnosis	adrenal (left)	4	[18m - 5y]	M	NA	NA	SCA	retroperitoneal (left)	Stroma-poor, poorly differentiated
TR2	Relapse	abdominopelvic	4S	< 18m	M	NA	NA	NCA	retroperitoneal	Neuroblastic tumor exhibiting post-
TR3	Relapse	retroperitoneal, pancreatic	4	> 5y	M	NA	NA	SCA	cervical lymphadenopathy	Stroma-poor, poorly differentiated
TR4	Relapse	pre-renal (right)	4	[18m - 5y]	F	NA	NA	SCA	temporal fossa (left)	Compatible with poorly differentiated
TR5	Relapse	adrenal (right)	Localized	[18m - 5y]	F	A	NA	SCA	lung	Compatible with a neuroblastoma.
TR6	Relapse	adrenal (right)	4S	< 18m	M	A	NA	SCA	iliac lymph node	Compatible with a stroma-poor, undifferentiated
TR7	Relapse	abdominal	4	[18m - 5y]	M	NA	NA	SCA	retroperitoineal (left)	Stroma-poor, poorly differentiated

m: months, y: years
A: amplified; NA: non amplified.
NCA: Numerical chromosome Aberrations; SCA: Segmental Chromosome Aberrations.
MKI: Mitosis-Karyorrhexis Index
*: NF1 context

Supplementary Table 2: Characteristics of the 15 neuroblastoma PDX models studied by scRNA-seq.

Name	Primary tumor at diagnosis	INSS Stage	Age at diagnosis	Sex	Status at biopsy for PDX	Site of biopsy	MYCN status	ALK status	Chr 1p deletion	Chr 17q gain
GR-NB4	Adrenal	4	[18m - 5y]	F	Relapse	Primary tumor	A	A	Yes	WG
GR-NB5	Adrenal	4	[18m - 5y]	M	Relapse	Lymph Node	A	WT	Yes	No
GR-NB7	Thoracic-abdominal	4	[18m - 5y]	M	Relapse	Bone	A	WT	Yes	Yes
GR-NB10	Adrenal	4	> 5y	M	Relapse	Bone	NA	WT	No	Yes
IC-pPDX-63	Adrenal	4	> 5y	M	Relapse	Frontal brain	A	WT	No	Yes
IC-pPDX-75	Thoracic	4	> 5y	F	Relapse	Liver	NA	F1174L	No	Yes
IC-pPDX-109	Abdominal	4	> 5y	M	Refractory	Primary tumor	A	WT	Yes	Yes
IC-pPDX-112	Adrenal	4	[18m - 5y]	M	Diagnosis	Primary tumor	A	WT**	Yes	Yes
IC-pPDX-196	Pre-renal	4	[18m - 5y]	F	Relapse	Temporal fossa	NA	WT	Yes	Yes
IC-pPDX-197	Adrenal	Localized	[18m - 5y]	F	Relapse	Lung	A	WT	Yes	Yes
HSJD-NB-003	Paravertebral	4	[18m - 5y]	F	Diagnosis	Liver	NA	WT	Yes	Yes
HSJD-NB-004*	Renal	4	[18m - 5y]	F	Diagnosis	Lymph Node	A	WT	No	No
HSJD-NB-005*	Renal	4	[18m - 5y]	F	Refractory	Bone Marrow	A	WT	No	Yes
HSJD-NB-009	Unknown	4	[18m - 5y]	M	Relapse	Bone	NA	E1419K	No	Yes
HSJD-NB-011	Retroperitoneal	4	[18m - 5y]	M	Relapse	Primary tumor	A	I1171N	Yes	Yes

* These two models have been generated from the same patient.
** Genomic amplification part of intron 3 of ALK gene.
m: months, y: years, A: amplified; NA: not amplified; WG: Whole Chromosome 17 gain

Supplementary Table 3: Filtering of human cells with high quality data for the scRNAseq done on the 18 neuroblastoma biopsies.

			Quality filters				Summary after filtering			
**Samples**	**nCells**	**global ambient mRNA contamination fraction**	**nDoublets**	**Coverage thresholds**	**minimum nGenes**	**%MT**	**nCells**	**%cells kept**	**median Cov**	**median nGenes**
**TR2**	4614	1.00%	584	[417, 36140]	316	20%	2518	55%	6926	2857.5
**TR3**	3183	1.72%	111	[426, 89556]	290	20%	2571	81%	2733	1338
**TR4**	10990	0.27%	206	[862, 46143]	501	20%	2407	22%	2398	1263
**TR5**	1434	0.87%	50	[424, 104973]	265	20%	1318	92%	4410.5	1989
**TR6**	3715	1.14%	220	[394, 123516]	217	20%	2519	68%	7071	2845
**TR7**	5113	1.00%	56	[406, 85880]	258	20%	4312	84%	8255.5	3225
**TD3**	936	0.79%	91	[501, 58534]	242	20%	768	82%	9177	3482
**TD4**	1725	1.00%	43	[453, 27085]	283	20%	1357	79%	2982	1544
**TD5**	2763	1.17%	129	[456, 88424]	301	20%	2258	82%	11940	3996
**TD6**	3651	0.07%	151	[461, 51657]	345	20%	3274	90%	7236.5	2946.5
**TD7**	1158	0.58%	0	[441, 84261]	295	20%	1083	94%	14357	4462
**TD8**	4734	0.03%	15	[408, 59874]	317	20%	3954	84%	8006	3305.5
**TD9**	4730	0.08%	22	[365, 81832]	229	20%	3284	69%	2634.5	1496
**TD10**	3793	1.00%	34	[565, 80782]	397	20%	3606	95%	9332.5	3543.5
**TD11**	1445	1.00%	0	[460, 130809]	276	20%	1305	90%	9695	3485
**TD13**	10898	1.00%	602	[403, 52270]	330	20%	9853	90%	5343	2525
**TD14**	3508	1.00%	57	[420, 61050]	276	20%	3234	92%	9521	3391
**TD15**	5878	3%	132	[419, 37194]	227	20%	4782	81%	2302.5	1093

Supplementary Table 4: Filtering of human cells with high quality data for the scRNAseq done on 15 neuroblastoma PDX models.

		Quality filters					Summary after filtering			
Samples	Retained Human/ human-murine doublet cells	filtered murine cells	Filtered murine cells (%)	Coverage thresholds	minimum nGenes	%MT	nCells	%cells kept	median Cov	median nGenes
**GR-NB4-p4**	7375	216	2.85%	[758, 33987]	465	20%	1106	15%	6330.5	2768.5
**GR-NB5-p5**	6396	62	0.96%	[1194, 31200]	714	20%	2344	37%	2074	1232.5
**GR-NB7-p5**	5711	1062	15.68%	[1012, 36064]	660	20%	2311	40%	2753	1674
**GR-NB10-p5**	4221	138	3.17%	[1183, 17057]	625	20%	518	12%	1949	1131.5
**HSJD-NB-003**	4567	778	14.56%	[347, 45359]	262	20%	3235	71%	12339	4205
**HSJD-NB-004**	8385	303	3.49%	[542, 15928]	376	20%	7069	84%	4996	2156
**HSJD-NB-005-pn***	7170	287	3.85%	[893, 22074]	620	20%	6705	94%	2909	1521
**HSJD-NB-005-pn+1***	7223	134	1.82%	[613, 31880]	397	20%	2090	29%	2776	1478.5
**HSJD-NB-009**	2678	1	0.04%	[484, 58234]	258	20%	1761	66%	13192	4488
**HSJD-NB-011**	7336	767	9.47%	[744, 24092]	461	20%	2114	29%	7064.5	2930.5
**IC-pPDX-63-p7***	5890	218	3.57%	[406, 49296]	321	20%	4267	72%	4952	2527
**IC-pPDX-63-p12***	5360	30	0.56%	[284, 4148]	222	20%	4265	80%	1104	728
**IC-pPDX-75-p5***	8480	148	1.72%	[876, 16969]	559	20%	887	10%	2411	1360
**IC-pPDX-75-p8***	4614	92	1.95%	[496, 38026]	351	20%	3246	70%	10470	3736
**IC-pPDX-109-p2**	4929	537	9.82%	[560, 39260]	391	20%	4590	93%	8612	3330
**IC-pPDX-112-p4**	6975	268	3.70%	[901, 21545]	619	20%	4870	70%	1748	1134
**IC-pPDX-196-p3**	5882	100	1.67%	[339,29319]	242	20%	2745	47%	2068	1238
**IC-pPDX-197-p4**	13620	118	0.86%	[1005,31892]	721	20%	11239	83%	4168	2184

* For these three models, two biological replicates have been done to assess reproducibility along passages in mice, however, only one of the replicates was included in the integration.
