# Supplementary material for: Reversible transitions between noradrenergic and mesenchymal tumor identities define cell plasticity in neuroblastoma
Source: Nat Commun. 2023 May 4;14:2575. doi: 10.1038/s41467-023-38239-5 (PMC10160107; doi:10.1038/s41467-023-38239-5)
Supplement: Supplementary file 2 — Description of additional supplementary files [file 41467_2023_38239_MOESM2_ESM.pdf]

<u>Description of additional supplementary files</u>

Supplementary Data 1: List of genes that are up-regulated in the different clusters of noradrenergic cells in a series of 18 neuroblastoma biopsies.

Supplementary Data 2: List of genes that are up-regulated in the different clusters of noradrenergic cells in a series of 15 neuroblastoma PDX models.
