# Supplementary material for: Reversible transitions between noradrenergic and mesenchymal tumor identities define cell plasticity in neuroblastoma
Source: Nat Commun. 2023 May 4;14:2575. doi: 10.1038/s41467-023-38239-5 (PMC10160107; doi:10.1038/s41467-023-38239-5)
Supplement: Supplementary file 4 — Reporting Summary [file 41467_2023_38239_MOESM4_ESM.pdf]

nature portfolio

Corresponding author(s): Isabelle Janoueix-Lerosey

Last updated by author(s): 2023/03/27

Reporting Summary

Nature Portfolio wishes to improve the reproducibility of the work that we publish. This form provides structure for consistency and transparency in reporting. For further information on Nature Portfolio policies, see our Editorial Policies and the Editorial Policy Checklist.

Statistics

For all statistical analyses, confirm that the following items are present in the figure legend, table legend, main text, or Methods section.

n/a	Confirmed	
☐	☒	The exact sample size (*n*) for each experimental group/condition, given as a discrete number and unit of measurement
☐	☒	A statement on whether measurements were taken from distinct samples or whether the same sample was measured repeatedly
☐	☒	The statistical test(s) used AND whether they are one- or two-sided<br>*Only common tests should be described solely by name; describe more complex techniques in the Methods section.*
☒	☐	A description of all covariates tested
☐	☒	A description of any assumptions or corrections, such as tests of normality and adjustment for multiple comparisons
☐	☒	A full description of the statistical parameters including central tendency (e.g. means) or other basic estimates (e.g. regression coefficient) AND variation (e.g. standard deviation) or associated estimates of uncertainty (e.g. confidence intervals)
☐	☒	For null hypothesis testing, the test statistic (e.g. *F*, *t*, *r*) with confidence intervals, effect sizes, degrees of freedom and *P* value noted<br>*Give P values as exact values whenever suitable.*
☒	☐	For Bayesian analysis, information on the choice of priors and Markov chain Monte Carlo settings
☒	☐	For hierarchical and complex designs, identification of the appropriate level for tests and full reporting of outcomes
☒	☐	Estimates of effect sizes (e.g. Cohen's *d*, Pearson's *r*), indicating how they were calculated

Our web collection on statistics for biologists contains articles on many of the points above.

Software and code

Policy information about availability of computer code

Data collection	We did not use any software for data collection.
Data analysis	For scRNA-seq analysis, we used:<br>- CellRanger 3.1.0 for demultiplexing and read mapping<br>- SoupX v1.4.5 for ambient RNA estimation and correction<br>- Scrublet v0.2.1 for doublets detection<br>- Seurat v3.1.5 for data filtering and normalization, Seurat v4.3.0 for data integration<br>- Harmony v1.0 for data integration<br>- InferCNV v1.9.1 to infer copy number variation<br>- R / RStudio (version 4.0.0) for single cell analyses and plots<br><br>For RNAseq analysis, we used:<br>- STAR 2.6.1a_08-27 for read mapping<br>- Cufflinks v2.2.1 for FPRM calculation<br>- R version 3.3.2 and Limma R package (version 3.30.7)for data normalization<br><br>For ChIPseq data analysis, we used:<br>- Bowtie2 v2.2.9 for read mapping<br>- Samtools v1.9 for filtering low quality reads<br>- HMCan v1.40 for peak calling<br>- LILY v1.1 for SE calling and valley detection: https://github.com/BoevaLab/LILY

FlowJo 9.9.6 for flow cytometry plotting.

GraphPad Prism 8 for graph representation and statistical analyses.

Mouse and human reference genomes are from the GRC website (https://www.ncbi.nlm.nih.gov/grc).

For manuscripts utilizing custom algorithms or software that are central to the research but not yet described in published literature, software must be made available to editors and reviewers. We strongly encourage code deposition in a community repository (e.g. GitHub). See the Nature Portfolio guidelines for submitting code & software for further information.

Data

Policy information about availability of data

All manuscripts must include a data availability statement. This statement should provide the following information, where applicable:

- Accession codes, unique identifiers, or web links for publicly available datasets
- A description of any restrictions on data availability
- For clinical datasets or third party data, please ensure that the statement adheres to our policy

All main and supplementary figures have associated raw data, now provided as a Source Data file.

Data availability:
Source data are provided with this paper as a Source Data file.
Alignment of sequencing data were realized using the hg19/GRCh37, the hg38/GRCh38 and the mm10/GRCh38 public databases of the GRC website (https://www.ncbi.nlm.nih.gov/grc).
ChIP-seq (RRID:SCR_001237) data of cell lines are available in Gene Expression Omnibus (GEO, RRID:SCR_005012) under the accession code GSE154907.
All single-cell RNA-seq from biopsies and PDXs, RNA-seq and the ChIP-seq data on the IC-pPDXC-63 cell line are available in European Genome-Phenome Archive (EGA) under the accession numbers EGAS00001004781 and EGAS00001005322. Patient tumor bulk RNAseq have already been published (EGAS00001005935).

Human research participants

Policy information about studies involving human research participants and Sex and Gender in Research.

Reporting on sex and gender	Sex of patients is mentioned in Tables S1 and S2, however, this information was not a criteria for study design or data interpretation.
Population characteristics	This research was not designed as a population study. Only 33 patient samples were profiled, 15 according to the PDX model generated. Whether patient tumors correspond to diagnosis or relapse is indicated, together with the following information: primary tumor site at diagnosis, INSS stage, age at diagnosis (< 18 months, between 18 months and 5 years, > 5 years-old), site of biopsy, sex and genetic alterations such as MYCN and ALK status.
Recruitment	Patients were not actively recruited for this study.
Ethics oversight	Written informed consents for the establishment of PDXs and/or for single-cell analyses were obtained for all patients from parents or guardians. Some correspond to patients enrolled in the MICCHADO study (ClinicalTrials.gov identifier NCT03496402) and/or in the MAPPYACTS trial (ClinicalTrials.gov identifier NCT02613962). Within these studies, approval of this research was given by the decision of the ethics committees Sud Est VI, reference AU 1388, and Ile de France III, reference Am7158-2-3272. The study was approved by the Institut Curie's Institutional Review Board (CRI-DATA220185).

Note that full information on the approval of the study protocol must also be provided in the manuscript.

Field-specific reporting

Please select the one below that is the best fit for your research. If you are not sure, read the appropriate sections before making your selection.

☒ Life sciences ☐ Behavioural & social sciences ☐ Ecological, evolutionary & environmental sciences

For a reference copy of the document with all sections, see nature.com/documents/nr-reporting-summary-flat.pdf

Life sciences study design

All studies must disclose on these points even when the disclosure is negative.

Sample size	For single-cell RNAseq, an input of 3,000-5,000 cells of each sample was loaded into the 10X Genomics Single-Cell Chromium Controller. These loading values were chosen to balance the probability of forming doublets with the aim of having sufficient recovery to obtain a complete overview of intra-tumor heterogeneity.

For FACS analysis, 20,000-100,000 viable cells were analyzed to obtain at least 10,000 cells to analyze for PHOX2B and CD44 expression.

Xenograft experiments included at least 6 mice per group to obtain statistical results. Sample size is included in the legend of the corresponding figure.

No statistical method was used to predetermine sample size.

Data exclusions	For single-cell RNAseq, low quality cells were filtered out based on a minimal number of UMIs and genes detected. We used various thresholds depending on the sample (see material and methods). We retained cells with at least 500 genes and 1,000 UMIs. Cells with >20% of UMIs coming from mitochondrial genes were removed.
Replication	Two biological replicates of scRNAseq were done for three models of PDX (HSLD-NB-005, IC-pPDX-63 and IC-pPDX75) at different passages in mice, which confirmed the reproducibility of tumor heterogeneity representation.<br>For FACS and IF, only one representative experiment out of 3 or more replicates is shown, when no quantification is needed but simply presence or absence of CD44 positive cells. All experiments gave the same conclusions. When quantification is needed to compare variation of CD44 positive cells or cell viability, the experiment counts 3 to 6 biological replicates to perform statistics, mentioned in the legends of the corresponding figures.
Randomization	We do not have experimental groups.
Blinding	We do not have experimental groups.

Behavioural & social sciences study design

All studies must disclose on these points even when the disclosure is negative.

Study description	*Briefly describe the study type including whether data are quantitative, qualitative, or mixed-methods (e.g. qualitative cross-sectional, quantitative experimental, mixed-methods case study).*
Research sample	*State the research sample (e.g. Harvard university undergraduates, villagers in rural India) and provide relevant demographic information (e.g. age, sex) and indicate whether the sample is representative. Provide a rationale for the study sample chosen. For studies involving existing datasets, please describe the dataset and source.*
Sampling strategy	*Describe the sampling procedure (e.g. random, snowball, stratified, convenience). Describe the statistical methods that were used to predetermine sample size OR if no sample-size calculation was performed, describe how sample sizes were chosen and provide a rationale for why these sample sizes are sufficient. For qualitative data, please indicate whether data saturation was considered, and what criteria were used to decide that no further sampling was needed.*
Data collection	*Provide details about the data collection procedure, including the instruments or devices used to record the data (e.g. pen and paper, computer, eye tracker, video or audio equipment) whether anyone was present besides the participant(s) and the researcher, and whether the researcher was blind to experimental condition and/or the study hypothesis during data collection.*
Timing	*Indicate the start and stop dates of data collection. If there is a gap between collection periods, state the dates for each sample cohort.*
Data exclusions	*If no data were excluded from the analyses, state so OR if data were excluded, provide the exact number of exclusions and the rationale behind them, indicating whether exclusion criteria were pre-established.*
Non-participation	*State how many participants dropped out/declined participation and the reason(s) given OR provide response rate OR state that no participants dropped out/declined participation.*
Randomization	*If participants were not allocated into experimental groups, state so OR describe how participants were allocated to groups, and if allocation was not random, describe how covariates were controlled.*

Ecological, evolutionary & environmental sciences study design

All studies must disclose on these points even when the disclosure is negative.

Study description	*Briefly describe the study. For quantitative data include treatment factors and interactions, design structure (e.g. factorial, nested, hierarchical), nature and number of experimental units and replicates.*
Research sample	*Describe the research sample (e.g. a group of tagged Passer domesticus, all Stenocereus thurberi within Organ Pipe Cactus National Monument), and provide a rationale for the sample choice. When relevant, describe the organism taxa, source, sex, age range and any manipulations. State what population the sample is meant to represent when applicable. For studies involving existing datasets, describe the data and its source.*
Sampling strategy	*Note the sampling procedure. Describe the statistical methods that were used to predetermine sample size OR if no sample-size calculation was performed, describe how sample sizes were chosen and provide a rationale for why these sample sizes are sufficient.*

March 2021

Data collection	*Describe the data collection procedure, including who recorded the data and how.*
Timing and spatial scale	*Indicate the start and stop dates of data collection, noting the frequency and periodicity of sampling and providing a rationale for these choices. If there is a gap between collection periods, state the dates for each sample cohort. Specify the spatial scale from which the data are taken*
Data exclusions	*If no data were excluded from the analyses, state so OR if data were excluded, describe the exclusions and the rationale behind them, indicating whether exclusion criteria were pre-established.*
Reproducibility	*Describe the measures taken to verify the reproducibility of experimental findings. For each experiment, note whether any attempts to repeat the experiment failed OR state that all attempts to repeat the experiment were successful.*
Randomization	*Describe how samples/organisms/participants were allocated into groups. If allocation was not random, describe how covariates were controlled. If this is not relevant to your study, explain why.*
Blinding	*Describe the extent of blinding used during data acquisition and analysis. If blinding was not possible, describe why OR explain why blinding was not relevant to your study.*

Did the study involve field work? ☐ Yes ☐ No

Field work, collection and transport

Field conditions	*Describe the study conditions for field work, providing relevant parameters (e.g. temperature, rainfall).*
Location	*State the location of the sampling or experiment, providing relevant parameters (e.g. latitude and longitude, elevation, water depth).*
Access & import/export	*Describe the efforts you have made to access habitats and to collect and import/export your samples in a responsible manner and in compliance with local, national and international laws, noting any permits that were obtained (give the name of the issuing authority, the date of issue, and any identifying information).*
Disturbance	*Describe any disturbance caused by the study and how it was minimized.*

Reporting for specific materials, systems and methods

We require information from authors about some types of materials, experimental systems and methods used in many studies. Here, indicate whether each material, system or method listed is relevant to your study. If you are not sure if a list item applies to your research, read the appropriate section before selecting a response.

Materials & experimental systems

n/a	Involved in the study	
☐	☒	Antibodies
☐	☒	Eukaryotic cell lines
☒	☐	Palaeontology and archaeology
☐	☒	Animals and other organisms
☒	☐	Clinical data
☒	☐	Dual use research of concern

Methods

n/a	Involved in the study	
☐	☒	ChIP-seq
☐	☒	Flow cytometry
☒	☐	MRI-based neuroimaging

Antibodies

Antibodies used

ChIP
H3K27ac: Anti-Histone H3 (acetyl K27) antibody / Abcam / ab4729 / GR32311937-1; GR3216173-1 - dilution: 1µg per IP

Immunohistochemistry
PHOX2B : Phox2b (B-11) / Santa Cruz / sc-376997 / B-11 / #E0118 - dilution: 1/200

Flow cytometry
PHOX2B : Mouse Monoclonal Anti-Human Phox2b [Clone B-11] - AlexaFluor® 647/ Santa Cruz / sc-376997 AF647 / B-11 / #A0218 - dilution: 1/100
CD44: FITC anti-human CD44 Antibody / Biolegend / 338804 / BJ18 / #B285080 - dilution: 1/100

Immunofluorescence
PHOX2B : Phox2b(B-11) / Santa Cruz / sc-376997 / B-11 / #E0118 - dilution: 1/100

	CD44: Rabbit anti-CD44/ PROTEINTECH / 15675-1-AP / # 00064224- dilution: 1/100<br>Anti-Mouse-Cy5: CY5 DONKEY IGG ANTI MOUSE IGG (H+L) / JACKSON IMMUNO/ 715-175-150 - dilution: 1/100<br>Anti-Rabbit-Cy3: CY3 DONKEY IGG ANTI RABBIT IGG (H+L) / JACKSON IMMUNO / 711-165-152 - dilution: 1/100
Validation	All the antibodies used in this study were validated for use in human specimens by the manufacturers.<br><br>ChIP<br>H3K27ac: Anti-Histone H3 (acetyl K27) antibody / Abcam / ChIP qualified (Manufacturer's website)<br><br>Immunohistochemistry<br>PHOX2B : Phox2b(B-11) / Santa Cruz / IHC qualified (Manufacturer's website)<br><br>Flow cytometry<br>PHOX2B : Mouse Monoclonal Anti-Human Phox2b [Clone B-11] - AlexaFluor® 647/ Santa Cruz / FACS qualified (Manufacturer's website)<br>CD44: FITC anti-human CD44 Antibody / Biolegend / 338804 / FACS qualified (Manufacturer's website)<br><br>Immunofluorescence<br>PHOX2B : Phox2b(B-11) / Santa Cruz / IF qualified (Manufacturer's website)<br>CD44: Rabbit anti-CD44/ PROTEINTECH / IF qualified (Manufacturer's website)<br>Anti-Mouse-Cy5: CY5 DONKEY IGG ANTI MOUSE IGG (H+L) / JACKSON IMMUNO/ IF qualified (Manufacturer's website)<br>Anti-Rabbit-Cy3: CY3 DONKEY IGG ANTI RABBIT IGG (H+L) / JACKSON IMMUNO / IF qualified (Manufacturer's website)

Eukaryotic cell lines

Policy information about cell lines and Sex and Gender in Research

Cell line source(s)	The SK-N-SH and the SH-SY5Y cell lines have been obtained from the ATCC. The IC-pPDXC-63 and IC-pPDXC-109 cell lines were generated in this study (from male patients).
Authentication	Cell line authentication was done by STR profiling with PowerPlex® 16 HS System from Promega and Cytoscan HD array (Affymetrix).
Mycoplasma contamination	Cells were monthly checked by qPCR (Venor® GeM qEP, Minerva biolabs®) for the absence of mycoplasma.
Commonly misidentified lines<br>(See ICLAC register)	No misidentified lines were used in this study.

Palaeontology and Archaeology

Specimen provenance	*Provide provenance information for specimens and describe permits that were obtained for the work (including the name of the issuing authority, the date of issue, and any identifying information). Permits should encompass collection and, where applicable, export.*
Specimen deposition	*Indicate where the specimens have been deposited to permit free access by other researchers.*
Dating methods	*If new dates are provided, describe how they were obtained (e.g. collection, storage, sample pretreatment and measurement), where they were obtained (i.e. lab name), the calibration program and the protocol for quality assurance OR state that no new dates are provided.*

☐ Tick this box to confirm that the raw and calibrated dates are available in the paper or in Supplementary Information.

Ethics oversight	*Identify the organization(s) that approved or provided guidance on the study protocol, OR state that no ethical approval or guidance was required and explain why not.*

Note that full information on the approval of the study protocol must also be provided in the manuscript.

Animals and other research organisms

Policy information about studies involving animals; ARRIVE guidelines recommended for reporting animal research, and Sex and Gender in Research

Laboratory animals	Xenografts experiments and PDXs were performed in female Swiss Nude adult mice (Charles River Laboratories, strain code 620). After transfer, mice were housed for 2-3 weeks for acclimation, in the animal facility, in a room with a 12h:12h light/dark cycle, a temperature of 22 +/- 2°C and a targeted hygrometry from 40 to 70%. At the time of injection or engraftment, the mice were at least 8 weeks-old.
Wild animals	This study did not involve wild animals.
Reporting on sex	Only female mice have been used.

Field-collected samples | This study did not involve field-collected samples.

Ethics oversight | PDXs models were generated at Institut Curie from patients under an Institutional-Review Board-approved protocol (OBS170323CPP ref3272; dossier No. 2015-A00464-45).
In vivo experiments for this study were performed in accordance with the recommendations of the European Community (2010/63/UE) for the care and use of laboratory animals. Experimental procedures were specifically approved by the ethics committee of the Institut Curie CEEA-IC #118 (Authorization APAFIS#11206-2017090816044613-v2 and APAFIS#34207-2021120215196250-v1 given by National Authority) in compliance with the international guidelines.

Note that full information on the approval of the study protocol must also be provided in the manuscript.

Clinical data

Policy information about clinical studies

All manuscripts should comply with the ICMJE guidelines for publication of clinical research and a completed CONSORT checklist must be included with all submissions.

Clinical trial registration | *Provide the trial registration number from ClinicalTrials.gov or an equivalent agency.*

Study protocol | *Note where the full trial protocol can be accessed OR if not available, explain why.*

Data collection | *Describe the settings and locales of data collection, noting the time periods of recruitment and data collection.*

Outcomes | *Describe how you pre-defined primary and secondary outcome measures and how you assessed these measures.*

Dual use research of concern

Policy information about dual use research of concern

Hazards

Could the accidental, deliberate or reckless misuse of agents or technologies generated in the work, or the application of information presented in the manuscript, pose a threat to:

No	Yes	
☐	☐	Public health
☐	☐	National security
☐	☐	Crops and/or livestock
☐	☐	Ecosystems
☐	☐	Any other significant area

Experiments of concern

Does the work involve any of these experiments of concern:

No	Yes	
☐	☐	Demonstrate how to render a vaccine ineffective
☐	☐	Confer resistance to therapeutically useful antibiotics or antiviral agents
☐	☐	Enhance the virulence of a pathogen or render a nonpathogen virulent
☐	☐	Increase transmissibility of a pathogen
☐	☐	Alter the host range of a pathogen
☐	☐	Enable evasion of diagnostic/detection modalities
☐	☐	Enable the weaponization of a biological agent or toxin
☐	☐	Any other potentially harmful combination of experiments and agents

ChIP-seq

Data deposition

☒ Confirm that both raw and final processed data have been deposited in a public database such as GEO.

☒ Confirm that you have deposited or provided access to graph files (e.g. BED files) for the called peaks.

Data access links
May remain private before publication. | ChIP-seq data are available in Gene Expression Omnibus (GEO) under accession number GSE154907.

Files in database submission | Processed files:

Files in database submission

SKNSH_CD44pos_H3K27ac_regions.bed
SKNSH_CD44neg_H3K27ac_regions.bed
SKNSHm_CD44pos_H3K27ac_regions.bed
SKNSHm_CD44neg_H3K27ac_regions.bed
xenoSHSY5Y_0_H3K27ac_regions.bed
xenoSHSY5Y_2_H3K27ac_regions.bed
xSKNSH_CD44pos_NSG4_H3K27ac_regions.bed
xSKNSH_CD44pos_NSG5_H3K27ac_regions.bed
xSKNSH_CD44neg_NSG2_H3K27ac_regions.bed
xSKNSH_CD44neg_NSG4_H3K27ac_regions.bed
SKNSH_CD44pos.H3K27ac.renorm.bw
SKNSH_CD44neg.H3K27ac.renorm.bw
SKNSHm_CD44pos_H3K27ac_regions.bed
SKNSHm_CD44neg_H3K27ac_regions.bed
xenoSHSY5Y_0.H3K27ac.renorm.bw
xenoSHSY5Y_2.H3K27ac.renorm.bw
xSKNSH_CD44pos_NSG4.H3K27ac.renorm.bw
xSKNSH_CD44pos_NSG5.H3K27ac.renorm.bw
xSKNSH_CD44neg_NSG2.H3K27ac.renorm.bw
xSKNSH_CD44neg_NSG4.H3K27ac.renorm.bw

Raw files:
A1131C08.R1.fastq.gz
A1131C05.R1.fastq.gz
A1131C04.R1.fastq.gz
A1131C01.R1.fastq.gz
D772C08.R1.fastq.gz
D772C05.R1.fastq.gz
D370C26.R1.fastq.gz
D370C25.R1.fastq.gz
D370C28.R1.fastq.gz
D370C27.R1.fastq.gz
D370C02.R1.fastq.gz
D370C01.R1.fastq.gz
D370C04.R1.fastq.gz
D370C03.R1.fastq.gz
D370C20.R1.fastq.gz
D370C19.R1.fastq.gz
D370C14.R1.fastq.gz
D370C13.R1.fastq.gz
D370C16.R1.fastq.gz
D370C15.R1.fastq.gz

Genome browser session
(e.g. UCSC)

The files provided in our GEO dataset can be easily downloaded in a genome browser session.

Methodology

Replicates

We have biological replicates for xenografts in this study (called rep 1 and rep 2).

Sequencing depth

Length of reads: 101
Reads are single-end.

Antibodies

We used the anti-histone H3 (acetyl K27) antibody, ChIP Grade (ab4729, rabbit polyclonal, Abcam).
https://www.abcam.com/histone-h3-acetyl-k27-antibody-chip-grade-ab4729.html
Lot numbers: GR32311937-1; GR3216173-1

Peak calling parameters

Reads were mapped on the indexed human reference genome hg19/GRCh37 using bowtie2 v2.2.9 with the following parameters --very-sensitive --end-to-end –reorder.
Command line: bowtie2 -x hg19 -U sample.fastq | samtools view -q 20 -bS - > sample.hg19.q20.bam
Peak calling was performed on quality-filtered.bam files using HMCan v1.40 with the following parameters on a matched pair of ChIP and Input samples:
GCIndex=GC_profile_25KbWindow_Mapp76_hg19.cnp
minLength=100
medLength=250
maxLength=400
smallBinLength=50
largeBinLength=25000
pvalueThreshold=0.05
mergeDistance=200
iterationThreshold=5
finalThreshold=0.1
maxIter=20
PosteriorProb=0.5

PrintWig=True
PrintPosterior=True
blackListFile=hg19-blacklist.bed
PrintBedGraph=True
CallPeaks=True
pairedEnds=False
RemoveDuplicates=False
HMCan command line: HMCan sample.hg19.q20.bam input.hg19.q20.bam config.txt sampleName
Regions from the hg19 ENCODE blacklist (Amemiya et al, 2019) were excluded from the analysis. Resulting peaks were quality-filtered using the LILY package while calling super-enhancers using log10-likelihood score of 5 (Boeva et al, 2017).
Amemiya HM, Kundaje A, Boyle AP. The ENCODE Blacklist: Identification of Problematic Regions of the Genome. Scientific Reports. 2019;9:1–5.
Boeva V, Louis-Brennetot C, Peltier A, Durand S, Pierre-Eugène C, Raynal V, et al. Heterogeneity of neuroblastoma cell identity defined by transcriptional circuitries. Nature Genetics. 2017;49:1408–13.

Data quality

Peaks were called using the HMCan v1.40 software. HMCan data normalization includes correction for the GC-content bias and bias due to copy number aberrations present in cancer samples. The ChIP signal in normalized by subtracting the Input signal with a constant representing the noise level; noise level varied from 30% to 50% in samples processed. HMCan calls peaks using hidden Markov models and calculates the log10 likelihood score. Within the LILY pipeline, peaks were filtered with log10-likelihood score of 5. This filtering removed from 40 to 60% of peaks called; the resulting number of peaks varied between 40,000 and 95,000.

Software

We used Bowtie2 v2.1.0 for read mapping (Langmead et al, 2012), (Samtools v1.9 for filtering low quality reads (Li H.*, Handsaker B.* et al, 2009), HMCan v1.40 for peak calling (Ashoor et al, 2013) and LILY v1.1 for SE calling and valley detection (Boeva et al, 2017; https://github.com/BoevaLab/LILY)

Langmead, B. & Salzberg, S. L. Fast gapped-read alignment with Bowtie 2. Nat. Methods 9, 357–359 (2012).
Li H, Handsaker B, Wysoker A, Fennell T, Ruan J, Homer N, et al. The Sequence Alignment/Map format and SAMtools. Bioinformatics. 2009;25:2078–9.
Ashoor H, Hérault A, Kamoun A, Radvanyi F, Bajic VB, Barillot E, et al. HMCan: a method for detecting chromatin modifications in cancer samples using ChIP-seq data. Bioinformatics. 2013;29:2979–86.
Boeva V, Louis-Brennetot C, Peltier A, Durand S, Pierre-Eugène C, Raynal V, et al. Heterogeneity of neuroblastoma cell identity defined by transcriptional circuitries. Nature Genetics. 2017;49:1408–13.

Flow Cytometry

Plots

Confirm that:

☒ The axis labels state the marker and fluorochrome used (e.g. CD4-FITC).

☒ The axis scales are clearly visible. Include numbers along axes only for bottom left plot of group (a 'group' is an analysis of identical markers).

☒ All plots are contour plots with outliers or pseudocolor plots.

☒ A numerical value for number of cells or percentage (with statistics) is provided.

Methodology

Sample preparation

For analysis:
Cells were detached with TrypLE™ Express Enzyme (Cat# 12604013, Gibco) and suspended in PBS. Cells were stained with Live/Dead fixable dead cell stain kit (Cat#L34955, Invitrogen) and permeabilized with the IntraPrep kit (Cat# A07803, BeckmanCoulter). The cell suspension was incubated with PHOX2B [Clone B-11] - AlexaFluor® 647 (Cat# SC-376997 AF647, Santa Cruz) and CD44-FITC (Cat# 338804, Biolegend) antibodies diluted at 1/100 during 30 min at 4°C in dark.

For sorting:
Flow cytometry sorting was performed with the SH800 cell sorter (Sony). Cells were detached with TrypLE™ Express Enzyme (Cat# 12604013, Gibco), suspended in PBS and incubated with CD44-FITC antibody diluted at 1/100 during 30 min at 4°C in dark. DAPI (Cat#62248, ThermoScientific) was used at 1/1000 to exclude dead/dying cells.

Instrument

The BD™ LSRII and Fortessa cytometer were used for analysis and the SH800 cell sorter (Sony) for cell sorting.

Software

FlowJo 9.9.6

Cell population abundance

For SK-N-SH and SK-N-SHm, after gating on live cells and on singulets, the CD44neg cells represents > 55% and the CD44pos cells >45%. CD44neg cells represent around 30% and CD44pos around 25% of the total ungated population.

For IC-pPDXC-63, after gating on live cells and on singulets, the CD44neg cells represent > 70% and the CD44neg cells >12%. CD44neg cells represent around 15% and CD44pos around 2.5% of the total ungated population.

The purity is estimated around 99.5% for CD44pos cells and 100% for CD44neg cells, and the sorting quality was checked after sorting on the same device.

Gating strategy

The first gating based on FSC/SSC represents 60% for IC-pPDXC-63 and 75% for SK-N-SH. Doublet cells are eliminated by gating on FSC-W / FSC- A followed by SSC-W / SSC-A. The second gating based on DAPI negative staining eliminates dying

cells. The boundaries between positive staining and negative staining are always more than 1 Log of fluorescence intensity. A control tube without staining is always analyzed to determine auto-fluorescence.

☒ Tick this box to confirm that a figure exemplifying the gating strategy is provided in the Supplementary Information.

Magnetic resonance imaging

Experimental design

Design type — *Indicate task or resting state; event-related or block design.*

Design specifications — *Specify the number of blocks, trials or experimental units per session and/or subject, and specify the length of each trial or block (if trials are blocked) and interval between trials.*

Behavioral performance measures — *State number and/or type of variables recorded (e.g. correct button press, response time) and what statistics were used to establish that the subjects were performing the task as expected (e.g. mean, range, and/or standard deviation across subjects).*

Acquisition

Imaging type(s) — *Specify: functional, structural, diffusion, perfusion.*

Field strength — *Specify in Tesla*

Sequence & imaging parameters — *Specify the pulse sequence type (gradient echo, spin echo, etc.), imaging type (EPI, spiral, etc.), field of view, matrix size, slice thickness, orientation and TE/TR/flip angle.*

Area of acquisition — *State whether a whole brain scan was used OR define the area of acquisition, describing how the region was determined.*

Diffusion MRI ☐ Used ☐ Not used

Preprocessing

Preprocessing software — *Provide detail on software version and revision number and on specific parameters (model/functions, brain extraction, segmentation, smoothing kernel size, etc.).*

Normalization — *If data were normalized/standardized, describe the approach(es): specify linear or non-linear and define image types used for transformation OR indicate that data were not normalized and explain rationale for lack of normalization.*

Normalization template — *Describe the template used for normalization/transformation, specifying subject space or group standardized space (e.g. original Talairach, MNI305, ICBM152) OR indicate that the data were not normalized.*

Noise and artifact removal — *Describe your procedure(s) for artifact and structured noise removal, specifying motion parameters, tissue signals and physiological signals (heart rate, respiration).*

Volume censoring — *Define your software and/or method and criteria for volume censoring, and state the extent of such censoring.*

Statistical modeling & inference

Model type and settings — *Specify type (mass univariate, multivariate, RSA, predictive, etc.) and describe essential details of the model at the first and second levels (e.g. fixed, random or mixed effects; drift or auto-correlation).*

Effect(s) tested — *Define precise effect in terms of the task or stimulus conditions instead of psychological concepts and indicate whether ANOVA or factorial designs were used.*

Specify type of analysis: ☐ Whole brain ☐ ROI-based ☐ Both

Statistic type for inference (See Eklund et al. 2016) — *Specify voxel-wise or cluster-wise and report all relevant parameters for cluster-wise methods.*

Correction — *Describe the type of correction and how it is obtained for multiple comparisons (e.g. FWE, FDR, permutation or Monte Carlo).*

Models & analysis

n/a	Involved in the study
☐	☐ Functional and/or effective connectivity
☐	☐ Graph analysis
☐	☐ Multivariate modeling or predictive analysis

Functional and/or effective connectivity	*Report the measures of dependence used and the model details (e.g. Pearson correlation, partial correlation, mutual information).*
Graph analysis	*Report the dependent variable and connectivity measure, specifying weighted graph or binarized graph, subject- or group-level, and the global and/or node summaries used (e.g. clustering coefficient, efficiency, etc.).*
Multivariate modeling and predictive analysis	*Specify independent variables, features extraction and dimension reduction, model, training and evaluation metrics.*
